# Microglia–astrocyte crosstalk as a key organizing principle of Alzheimer’s disease: from homeostatic cooperation to maladaptive signaling loops

**DOI:** 10.3389/fnagi.2026.1908574

**Published:** 2026-08-04

**Authors:** Mingqiang Gong, Jiao Lan, Jifei Miao

**Affiliations:** Shenzhen Bao’an Chinese Medicine Hospital, Guangzhou University of Chinese Medicine, Shenzhen, Guangdong, China

**Keywords:** Alzheimer’s disease, amyloid-beta, glial phenotypic heterogeneity, microglia–astrocyte crosstalk, neuroinflammation

## Abstract

Alzheimer’s disease (AD) has long been framed around amyloid-beta (Aβ) and tau pathology, yet mounting evidence indicates that dysfunctional microglia–astrocyte crosstalk is an important, and often underappreciated, contributor to disease progression that operates alongside—rather than in place of—neuronal, vascular, and proteinopathic mechanisms. Here we propose a three-stage framework in which glial communication transitions from silent vulnerability through organized defense to maladaptive collapse. During preclinical aging, gut dysbiosis, diminished tryptophan-derived aryl hydrocarbon receptor (AHR) ligands, and blood–brain barrier weakening prime glia toward inflammatory states with elevated complement tone. Upon Aβ accumulation, microglia and astrocytes initially mount a compensatory response—forming reactive glial nets, containing plaques, clearing tau, and executing complement-guided synaptic pruning. However, sustained pathological burden triggers self-reinforcing loops involving the C3–C3aR axis and IL-1α/TNF-α/C1q signaling, converting the glial network into a propagation engine for tau spreading and synapse loss—the strongest correlate of cognitive decline. We discuss the tryptophan–microbiota–AHR axis as one candidate upstream modulator, while emphasizing that direct human evidence remains limited, and highlight APOE4 in exacerbating microglia-dependent synaptic phagocytosis. To support testability, we operationally define maladaptive loops and communication collapse, and specify measurable variables, fluid/imaging biomarker proxies (e.g., the sTREM2/GFAP ratio), and falsifiable predictions. This framework, presented as an integrative hypothesis rather than an established principle, argues that future therapies must combine protein-targeted approaches with restoration of glial communication homeostasis.

## Graphical Abstract

**Figure:**
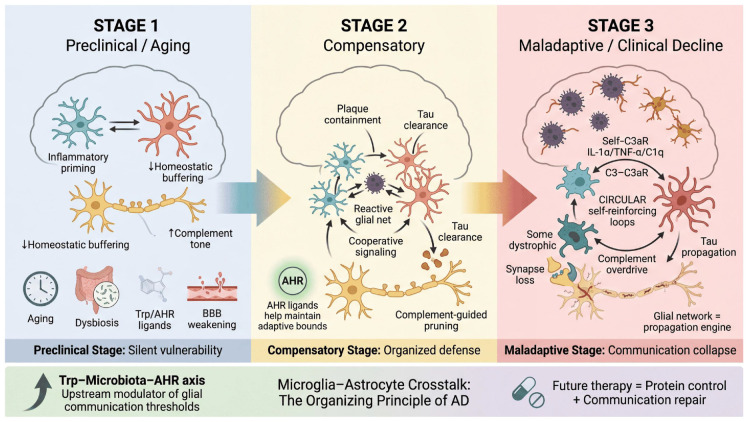
Microglia–astrocyte crosstalk: the organizing principle of Alzheimer’s disease. Alzheimer’s disease progresses through three stages of glial communication: silent vulnerability (aging, dysbiosis, reduced AHR ligands), organized compensatory defense (reactive glial nets containing plaques and tau), and maladaptive collapse (self-reinforcing C3–C3aR and IL-1α/TNF-α/C1q loops driving synapse loss). The tryptophan–microbiota–AHR axis tunes these thresholds throughout.

## 1 Introduction

Alzheimer’s disease (AD) is the most prevalent cause of dementia and represents one of the greatest biomedical challenges of an aging society (Soria Lopez et al., 2019; Jack et al., 2024). Clinically, AD manifests as a progressive decline in memory, executive function, language, and behavioral regulation; pathologically, it is defined by extracellular Aβ deposition and intracellular neurofibrillary tangles (NFTs) composed of hyperphosphorylated tau (Edler et al., 2023; Deng et al., 2024). Yet the persistence of therapeutic failure despite decades of effort indicates that the dominant pathological lesions do not, by themselves, fully explain disease progression. Importantly, the more recent anti-Aβ antibodies represent a genuine, mechanistically informative advance: lecanemab and donanemab both robustly lower amyloid burden and produce statistically significant, if clinically modest, slowing of decline in early symptomatic disease, providing the first prospective, interventional evidence that Aβ is causally upstream of clinical progression (Reardon, 2023; Söderberg et al., 2023). At the same time, the residual decline that persists despite near-complete plaque removal, and the stage-dependence of benefit, indicate that Aβ is a necessary and causally active—but not, by itself, sufficient—driver of disease once symptoms are established. These observations motivate, rather than contradict, the search for additional parallel mechanisms that determine how a given amyloid burden is translated into tissue dysfunction.

One reason for this insufficiency is that the disease has too often been modeled as a largely neuron-autonomous process. That view is increasingly untenable. Neurons do not exist in isolation; they operate within a highly integrated multicellular environment in which astrocytes, microglia, oligodendrocytes, endothelial cells, pericytes, and infiltrating immune cells together determine whether the tissue remains resilient or becomes permissive to degeneration (Javanmehr et al., 2022; Labarta-Bajo and Allen, 2025; Škandík et al., 2025). Within this cellular ecosystem, the interaction between microglia and astrocytes occupies a particularly strategic position. These two glial populations govern immune surveillance, phagocytosis, synaptic remodeling, trophic support, complement activation, lipid handling, blood–brain barrier (BBB) stability, and the neurochemical environment surrounding neurons (Tani et al., 2014; Vainchtein and Molofsky, 2020; Segarra et al., 2021; Wu and Eisel, 2023). Their crosstalk therefore does not merely accompany pathology; it shapes the conditions under which pathology becomes harmful.

A second reason lies in the failure of binary classifications that have long dominated glial biology. Microglia have often been divided into “M1” and “M2” states, and astrocytes into “A1” and “A2” phenotypes (Saijo and Glass, 2011; Boche et al., 2013). These heuristics were useful as early conceptual scaffolds, but they now obscure more than they clarify. Single-cell and single-nucleus transcriptomics have revealed extensive spatial and temporal heterogeneity in both cell types, including disease-associated microglia (DAM), amyloid-responsive microglia, dystrophic and terminally inflammatory microglia, as well as disease-associated astrocytes (DAAs) and multiple regionally constrained reactive states (Mucke et al., 2000; Liu W. et al., 2011; Zhang et al., 2025). These states are dynamic, context-dependent, and only partly captured by conventional inflammatory labels. More importantly, microglial and astrocytic phenotypes are not generated independently. Each is shaped by signals from the other.

This Review is built around a central proposition: microglia–astrocyte crosstalk is not merely a secondary consequence of AD but a key organizing axis that shapes how neuronal, vascular, and proteinopathic pathologies are translated into tissue dysfunction. We stress at the outset that we do not claim glial crosstalk to be the sole or dominant driver of AD; rather, we argue that it is one critical, and comparatively understudied, node within a multifactorial disease process. We argue that Aβ deposition, tau propagation, complement activation, synapse loss, and network dysfunction may become more clinically consequential when the homeostatic conversation between microglia and astrocytes is progressively distorted by maladaptive signaling loops. We further consider whether the tryptophan (Trp)–microbiota–aryl hydrocarbon receptor (AHR) axis may act as one upstream modulator of this transition. By integrating dietary input, intestinal microbial metabolism, host immunity, and glial transcriptional programs, this axis could provide a candidate mechanistic bridge between the microbiota–gut–brain axis (MGBA) and the central nervous system (CNS) pathology of AD. We stress at the outset, however, that the evidence supporting an upstream role for this axis is largely indirect and derived from separate experimental systems, that AHR signaling is markedly context-dependent and bidirectional, and that its relevance to human AD—and its therapeutic potential—remains uncertain. We therefore present it as one of several modulatory inputs worth testing, rather than as an established or dominant regulator.

This emphasis on glial communication is not meant to diminish the well-established contributions of other disease mechanisms. Neuron-autonomous processes—including impaired proteostasis, mitochondrial and metabolic dysfunction, and intrinsic vulnerability of selected neuronal populations—remain fundamental to AD, as does cerebrovascular pathology, encompassing blood–brain barrier breakdown, impaired perfusion, and cerebral amyloid angiopathy (De Strooper and Karran, 2016; Sweeney et al., 2018; Knopman et al., 2021). Equally fundamental are neuronal network–level mechanisms: neuronal and circuit hyperexcitability, disrupted excitation–inhibition balance, aberrant oscillatory and default-mode network activity, and activity-dependent facilitation of Aβ and tau release together link molecular pathology to the network dysfunction that tracks cognitive decline (Palop and Mucke, 2016; Busche and Hyman, 2020). Our framework is intended to complement these models—glial mismanagement of glutamate, complement, and synapse elimination is one plausible interface through which network hyperactivity and proteinopathy become mutually reinforcing—rather than to compete with them. AD is best understood as a multifactorial disorder in which these mechanisms act in parallel and interact reciprocally. Our aim is to argue that microglia–astrocyte crosstalk represents one particularly influential and integrative layer within this network—a layer that determines, in part, whether neuronal, vascular, and proteinopathic insults are contained or amplified—rather than to propose that it operates in isolation or supersedes them. We explicitly reject a dichotomy between “proteinopathy versus glia” or “vascular versus glia”: in our reading the evidence favors an integrated model in which amyloid and tau initiate and sustain pathology, neuronal networks propagate and register its functional cost, vascular dysfunction lowers tissue resilience, and glial crosstalk acts as a convergent node that modulates all three. Throughout this review we are careful to distinguish the level of evidence supporting each claim: findings derived from human tissue are largely associative and cross-sectional, whereas evidence for a causal, upstream role of disrupted crosstalk derives almost entirely from animal and *in vitro* models. Where we describe glial signals as “driving” or “promoting” pathology, this causal language reflects experimental (predominantly rodent) manipulation and should not be read as established causation in the human disease.

Our goal is therefore not to restate that microglia and astrocytes are “important” in AD—this is already well-established—but to develop a more precise and more provocative framework. Specifically, we examine how glial crosstalk operates in health, how it is progressively remodeled by aging, how it shapes Aβ and tau pathology, and why its regulatory logic breaks down in AD. Along the way, we highlight several unresolved tensions that, in our view, should become focal points for the next generation of AD research: Why do protective glial responses become harmful? Why does AHR signaling appear anti-inflammatory in one setting and pathogenic in another? Why do anti-inflammatory therapies often underperform despite strong evidence of neuroinflammation? And why does reducing Aβ burden fail to normalize tissue biology once glial communication has entered a self-sustaining pathological state?

## 2 Why microglia–astrocyte crosstalk is a central node in AD pathogenesis

The healthy adult brain is maintained by a dense web of reciprocal signaling among neurons, glial cells, and vascular elements (Segarra et al., 2021). Neurons depend on astrocytes for glutamate uptake, ion buffering, metabolic support, and regulation of the synaptic microenvironment (Tani et al., 2014; Lines et al., 2020). Microglia continually patrol the parenchyma, eliminate apoptotic debris, refine synapses, respond to damage-associated molecular patterns (DAMPs), and shape neuronal circuitry through trophic and immune mediators (Dejanovic et al., 2022; Scott-Hewitt et al., 2024). Endothelial cells and pericytes, in turn, regulate BBB integrity and neurovascular coupling, while oligodendrocytes sustain axonal metabolism and white matter stability (Abbott et al., 2006; Philips et al., 2021). Homeostasis emerges from communication within this cellular consortium.

Within this multicellular framework, microglia and astrocytes are uniquely positioned to coordinate tissue responses. Microglia are the principal innate immune sentinels of the CNS (Nimmerjahn et al., 2005; Liddelow et al., 2020). They are rapidly activated by infection, injury, Aβ species, extracellular ATP, and various forms of metabolic stress (Mizuno, 2012; Rodríguez-Gómez et al., 2020). Astrocytes, by contrast, are the most abundant glial population and serve as regional integrators of metabolic, vascular, and synaptic information (Emsley and Macklis, 2006; Sofroniew and Vinters, 2010; Pekny and Pekna, 2014). Their perisynaptic processes and vascular endfeet place them at the intersection of neuronal activity, BBB function, and inflammatory signaling. Because microglia respond earlier to perturbations and astrocytes exert broader control over tissue physiology, their interaction effectively determines whether local stress is contained, amplified, or translated into long-range pathology.

This relationship is not optional. Under physiological conditions, astrocytes provide trophic support for microglial survival and identity. Astrocyte-derived CSF-1, IL-34, TGF-β2, and cholesterol contribute to maintaining microglial populations and functional competence (Greter et al., 2012; Wang et al., 2012; Bohlen et al., 2017). Endothelial support, including heparin-binding EGF-like growth factor (HBEGF), also stabilizes astrocytic positioning and function near vascular structures (Foo et al., 2011). Conversely, microglia regulate astrocytic behavior through a wide range of cytokines, chemokines, growth factors, reactive oxygen species (ROS), nitric oxide (NO), extracellular vesicles (EVs), and complement components (Goetzl et al., 2016; Paolicelli et al., 2019; d’Errico et al., 2022). The physiological meaning of glial crosstalk, therefore, is not simply that one cell type “activates” another. Rather, both cell types participate in a tightly calibrated feedback system that balances surveillance, repair, synaptic plasticity, and immune restraint.

This is precisely why the breakdown of glial crosstalk is so consequential in AD. Once the communicative architecture between microglia and astrocytes becomes distorted, multiple downstream pathologies can emerge simultaneously: excessive complement deposition, maladaptive phagocytosis, impaired clearance of aggregated proteins, chronic cytokine amplification, altered lipid handling, vascular instability, and synaptic attrition. What appears superficially as a collection of parallel disease mechanisms may in fact represent different outputs of one progressively disordered communication network. We note, however, that this integrative reading does not subsume vascular or neuronal pathology into glial biology. Blood–brain barrier breakdown, impaired perfusion, and cerebral amyloid angiopathy can injure tissue through glia-independent routes (Sweeney et al., 2018; Knopman et al., 2021), and astrocytic endfeet and perivascular microglia are better viewed as one interface between the neurovascular unit and glial signaling than as the sole mediators of vascular contributions to AD.

A growing body of human and experimental evidence supports this view. Proteomic analyses of AD-related mouse models revealed that astrocyte-specific and microglia-specific proteins are unusually tightly connected in disease-associated co-expression networks, whereas this interaction is significantly reduced in human mild cognitive impairment, suggesting early disruption of glial cooperation (Liu et al., 2024). While consistent with early disruption of glial cooperation, these co-expression relationships are correlative and cannot, on their own, establish that altered glial coupling precedes or causes downstream pathology. Spatial transcriptomic studies further show that activated microglia, reactive astrocytes, and vulnerable neurons occupy structured microenvironments rather than random patterns of coexistence (Cain et al., 2023; Gabitto et al., 2024; Green et al., 2024). Such findings are consistent with the possibility that the tissue context of AD is shaped in part by cell–cell interactions, not merely by the cell-autonomous burden of Aβ and tau, though as cross-sectional observations they establish spatial association rather than causal ordering.

The conceptual implication is important. If microglia–astrocyte crosstalk is an important shaper of disease architecture, then therapeutic success is likely to depend not only on reducing pathological protein load, but also on restoring the logic of intercellular communication. This inference, however, remains provisional: the causal direction linking glial communication states to tissue outcomes has been demonstrated chiefly through manipulation in experimental models and awaits confirmation in humans. This shift from a lesion-centered to a communication-centered framework may help explain why interventions that improve one molecular lesion fail to reestablish tissue resilience once the glial network has already entered a maladaptive state.

## 3 Homeostatic microglia–astrocyte communication in the healthy CNS

To understand how glial crosstalk becomes pathogenic in AD, it is essential first to define what it accomplishes in the healthy brain. Homeostatic communication between microglia and astrocytes is multidimensional. It includes soluble mediators, receptor–ligand interactions, extracellular vesicles, direct membrane contacts, and the coordinated interpretation of neuronal and vascular signals (Rostami et al., 2021; Chakraborty et al., 2023).

At the synaptic level, astrocytes and microglia act as complementary custodians. Astrocytes take up synaptically released glutamate via transporters such as GLT-1/EAAT2, convert it within the glutamate–glutamine cycle, and thereby prevent excitotoxicity while supporting neurotransmitter recycling (Tani et al., 2014; Brandebura et al., 2023). They also respond to ATP, norepinephrine, and local circuit activity with calcium transients and gliotransmitter release, modulating synaptic efficacy and excitability. Microglia, in parallel, monitor synaptic integrity through pathways including CX3CL1–CX3CR1 signaling and complement-dependent recognition, contributing to the maintenance of stable yet plastic neural circuits (Pawelec et al., 2020; Cornell et al., 2022).

At the developmental and circuit refinement level, the cooperation between these glial populations is even clearer. Astrocytes engulf excess excitatory synapses through the MEGF10 and MERTK pathways (Chung et al., 2013), while microglia prune complement-tagged synapses through CR3-dependent mechanisms (Shi et al., 2015; Anderson et al., 2019). These are not redundant functions. They represent distributed labor within a shared surveillance system. Together, they allow the brain to eliminate weak or unnecessary synaptic connections while preserving active ones (Fonseca et al., 2017; Datta et al., 2020).

At the immune level, microglia function as the primary detectors of pathogens and DAMPs, owing in part to their broader repertoire of Toll-like receptors (TLRs) (Rodríguez-Gómez et al., 2020). Astrocytes express a more limited TLR profile and may be less capable of mounting primary pathogen-sensing responses on their own (Li et al., 2021; Garland et al., 2022). This asymmetry gives microglia a temporal advantage during acute disturbances. Yet astrocytes are not passive followers. Once engaged, they profoundly influence the duration, spread, and tissue consequences of inflammatory signaling by producing cytokines, chemokines, growth factors, metabolic mediators, and extracellular matrix components (Verkhratsky and Nedergaard, 2018; Matejuk and Ransohoff, 2020). In practical terms, microglia often initiate a local alert, but astrocytes determine whether that alert remains local, becomes reparative, or evolves into tissue-wide dysfunction.

**FIGURE 1:**
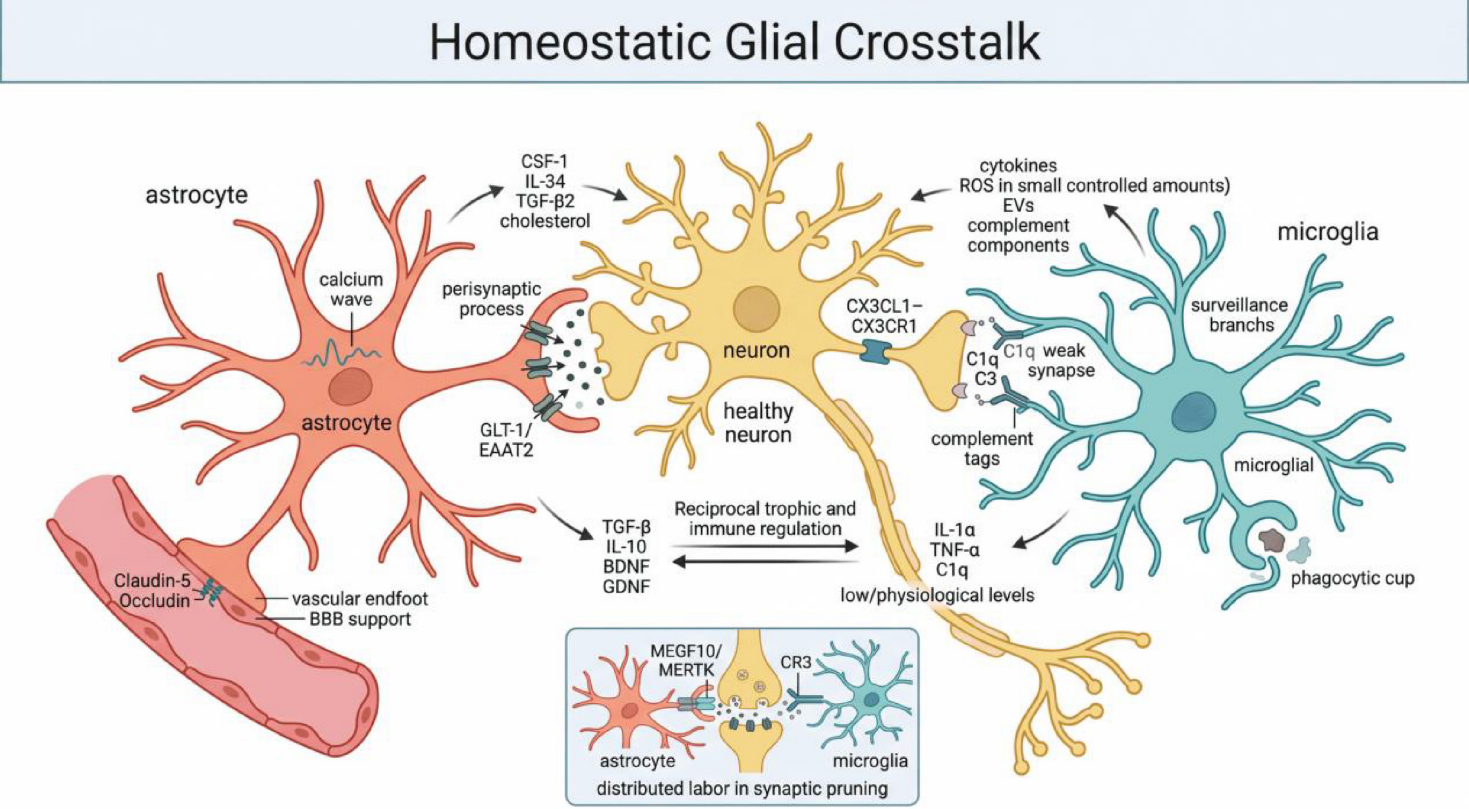
Homeostatic microglia–astrocyte crosstalk in the healthy central nervous system (CNS). Astrocytes support neuronal function through glutamate uptake [glutamate transporter 1 (GLT-1)/excitatory amino acid transporter 2 (EAAT2)], calcium signaling, and blood–brain barrier (BBB) maintenance via vascular endfeet expressing Claudin-5 and Occludin. They provide trophic factors (CSF-1, IL-34, TGF-β2, cholesterol) to microglia and regulatory mediators transforming growth factor beta (TGF-ß), interleukin-10 (IL-10), BDNF, GDNF. Microglia perform immune surveillance, monitor synaptic integrity via CX3CL1–CX3CR1 signaling, and release cytokines, extracellular vesicles (EVs), and complement components at physiological levels. Complement tags (C1q, C3) mark weak synapses for elimination. Inset: distributed labor in synaptic pruning—astrocytes via multiple EGF-like domains 10 (MEGF10)/MER proto-oncogene tyrosine kinase (MERTK), microglia via CR3.

At the vascular and metabolic level, the partnership is equally fundamental. Astrocytes, through their endfeet, help maintain BBB function and neurovascular coupling (Abbott et al., 2006; Tyurikova et al., 2025). Microglia interact with endothelial and perivascular cells to monitor vascular integrity and to clear debris or altered cells that threaten tissue homeostasis (Abbott et al., 2006; Munro et al., 2024). When functioning properly, the microglia–astrocyte partnership thus supports a coherent “operating environment” for neurons, one in which inflammatory vigilance, metabolic support, and structural maintenance are tightly integrated. Several lines of evidence further indicate that this partnership begins early and persists lifelong. Bidirectional glial communication operates not only in pathological states but in development, maturation, and healthy aging (Sun et al., 2023; Figure 1). Aging, however, gradually alters the basal set points of this interaction. The critical transition in AD is therefore not the sudden appearance of an entirely new signaling system, but the distortion of a physiological communication network that has become increasingly vulnerable over time.

## 4 Aging as the precondition for pathological crosstalk

Aging is the most important risk factor for AD (Barnes and Yaffe, 2011), yet it is often treated merely as a background variable rather than an active biological process that shapes disease susceptibility. This underestimates its mechanistic significance. Aging remodels the CNS at multiple levels: it alters cellular composition, weakens metabolic and vascular support, changes cytokine tone, impairs proteostatic capacity, and shifts the transcriptomic states of glia toward inflammatory priming (Li et al., 2024; Jin et al., 2025). In effect, aging lowers the threshold at which compensatory glial communication becomes maladaptive.

Microglia are among the most age-sensitive cells in the brain. Aged microglia show enhanced expression of pro-inflammatory cytokines, dysregulated phagocytosis, increased interferon and complement signaling, and a “primed” phenotype characterized by exaggerated responses to subsequent stressors (Olah et al., 2018; Pan et al., 2025). Morphologically, they can exhibit dystrophic features, and functionally they often lose the precise discrimination required for efficient clearance without collateral damage (Perry et al., 1993; Streit et al., 2004). In AD-prone contexts, such changes may predispose microglia to overreact to Aβ and tau while underperforming in their degradative tasks.

Astrocytes also undergo profound aging-associated remodeling. Aging pushes astrocytes away from homeostatic programs and toward reactive states characterized by elevated C3, Serpina3n, and inflammatory mediators, along with reduced expression of genes involved in water balance, glutamate handling, and neurovascular support (Liddelow et al., 2017; Brandebura et al., 2023). Astrocytic endfeet coverage declines, AQP4 and Kir4.1 distribution becomes disorganized, and BBB support weakens (Kiss et al., 2020; Pan et al., 2021; Tyurikova et al., 2025). These changes are not epiphenomena. They mean that the tissue matrix within which neurons and microglia operate becomes less buffered, more inflammatory, and more vulnerable to both peripheral influences and local excitotoxicity.

The neurovascular unit is a major casualty of aging. Endothelial cells reduce tight junction proteins such as Claudin-5 and Occludin while increasing ICAM1 and VCAM1, promoting BBB leakiness and peripheral immune cell entry (Kiss et al., 2020; Pan et al., 2021). Waste clearance and nutrient delivery become less efficient, particularly in hippocampal and deep cortical regions that are selectively vulnerable in cognitive decline (Smith et al., 2015; Zhang et al., 2019). Such vascular compromise changes the informational landscape of the brain. Signals that would once have remained compartmentalized can now diffuse more widely, and peripheral inflammatory or metabolic disturbances can increasingly influence central glial behavior.

Aging also affects synaptic and myelin homeostasis. Microglia aberrantly reactivate complement-mediated pruning programs, leading to the engulfment of structurally intact but vulnerable synapses marked by C1q and C3 (Dejanovic et al., 2022; Scott-Hewitt et al., 2024). Astrocytes show reduced GLT-1/EAAT2 expression and impaired release of modulators such as D-serine, compromising long-term potentiation and circuit stability (Brandebura et al., 2023). Oligodendrocyte progenitor cells (OPCs) lose regenerative competence, while aged microglia secrete factors that impair OPC differentiation and support white matter degeneration (Santos and Fields, 2021; Gomez et al., 2024; Li et al., 2024). These events broaden the consequences of glial crosstalk failure beyond plaque and tangle pathology.

**FIGURE 2:**
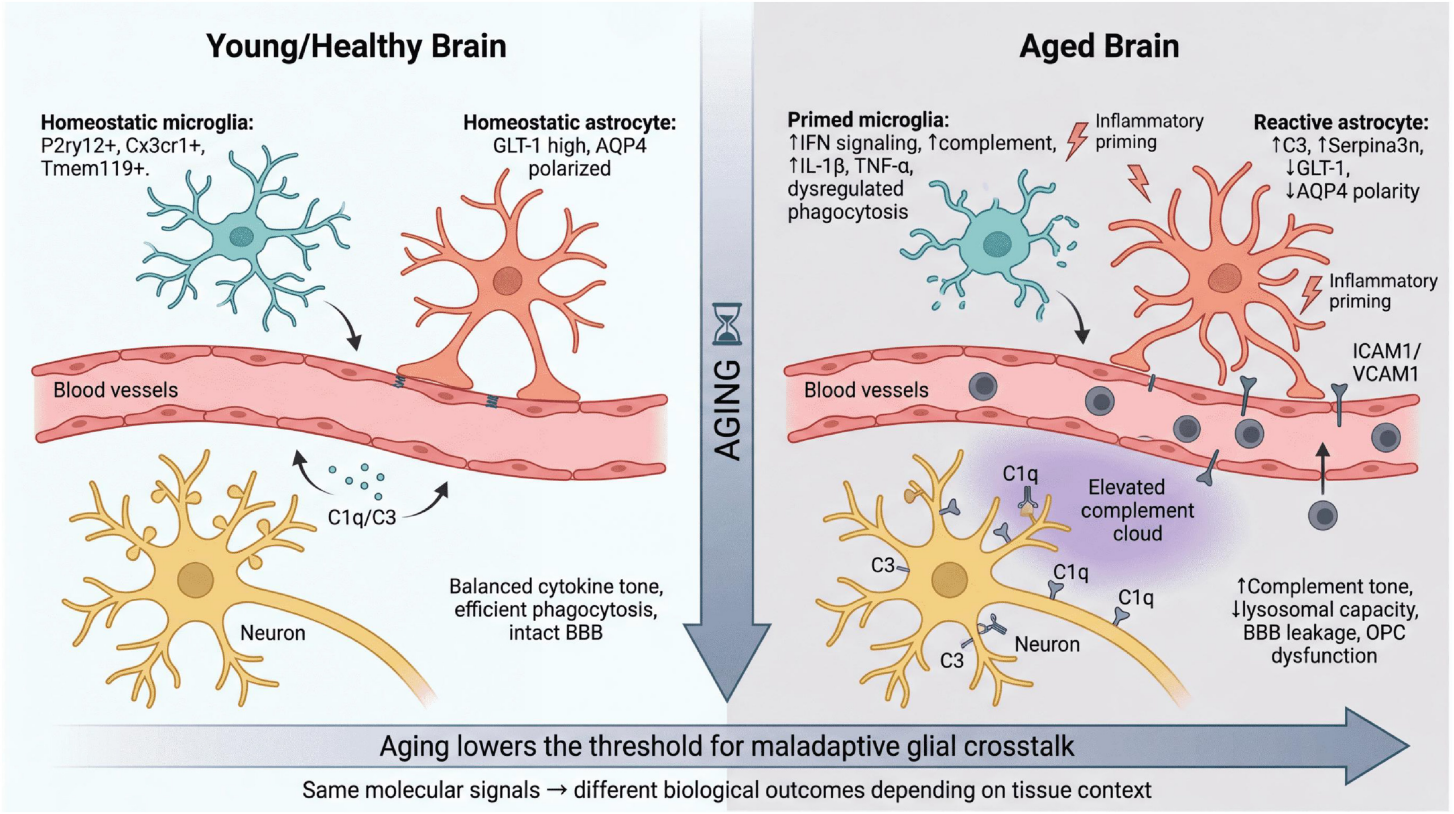
Aging remodels microglia–astrocyte crosstalk, lowering the threshold for maladaptive signaling. In the young brain (left), homeostatic microglia (P2ry12^+^, Cx3cr1^+^, Tmem119^+^) and astrocytes [glutamate transporter 1 (GLT-1) high, AQP4 polarized] maintain balanced cytokine tone, efficient phagocytosis, and intact blood–brain barrier (BBB). In the aged brain (right), microglia become primed (↑IFN, ↑complement, ↑IL-1β/TNF-α, dysregulated phagocytosis), while astrocytes shift toward reactive states (↑C3, ↑Serpina3n, ↓GLT-1, ↓AQP4 polarity). BBB integrity declines with increased ICAM1/VCAM1, and elevated complement deposition (C1q, C3) accumulates around synapses. Aging thus changes the biological meaning of identical intercellular signals depending on tissue context.

Taken together, aging does not merely increase the burden of “background inflammation.” It changes the meaning of intercellular signals. A cytokine, complement component, or AHR ligand delivered into a young, metabolically resilient tissue does not necessarily produce the same outcome in an aged brain already primed by inflammaging, vascular leakiness, and impaired lysosomal function. This point is essential for understanding why identical molecular pathways can appear protective in one experimental context and damaging in another (Figure 2).

## 5 Rethinking glial states in AD: beyond M1/M2 and A1/A2

Before examining specific disease mechanisms, it is necessary to address the conceptual limits of the traditional inflammatory classifications of microglia and astrocytes. The M1/M2 scheme for microglia and A1/A2 framework for astrocytes have provided accessible entry points into glial biology, but they are increasingly inadequate for describing disease reality (Escartin et al., 2021). The problem is not simply that these categories are “simplified”; it is that they imply stable and internally coherent states where there are often only partial, overlapping, and transitionary programs.

Microglia were historically divided into classically activated M1 cells, associated with pro-inflammatory cytokines such as IL-1β, IL-6, TNF-α, NO, and ROS, and alternatively activated M2 cells, associated with phagocytosis, tissue repair, and trophic factors including IGF-1, BDNF, TGF-β, and VEGF. This dichotomy captured a real tension between inflammatory injury and repair. Yet *in vivo* studies have repeatedly shown that microglia can express both M1- and M2-associated markers within the same cell, at the same time, and in a context-dependent manner (Millet et al., 2024; Flury et al., 2025). Furthermore, not all ostensibly “M2-like” functions are beneficial. IL-4-driven responses, for example, have been linked in some models to increased Aβ deposition, presumably through impaired clearance despite a less overtly inflammatory phenotype (Kiyota et al., 2010; Chakrabarty et al., 2012).

Recent single-cell studies have replaced this simplistic model with a much richer landscape. DAMs, first described in neurodegenerative conditions including AD, exhibit upregulation of genes such as Apoe, Trem2, Tyrobp, Lpl, and Ctsd, together with downregulation of homeostatic markers like P2ry12, Cx3cr1, Csf1r, and Tmem119 (Deczkowska et al., 2018; Hansen et al., 2018; Gabandé-Rodríguez et al., 2020; Wang, 2021). Amyloid-responsive microglia (ARM), dystrophic microglia, stress-associated microglia, and terminally inflammatory microglia (TIM) further expand this taxonomy (Nguyen et al., 2020). These states differ in metabolic wiring, phagocytic competence, inflammatory tone, and anatomical distribution. Some are likely adaptive attempts to contain pathology, while others may represent exhausted or maladaptive endpoints.

Beyond these amyloid-centered states, single-cell and spatial studies have defined additional microglial populations whose distinct anatomical and functional profiles are directly relevant to a stage-based model. White matter-associated microglia (WAM), which depend partly on TREM2 signaling and cluster around degenerating myelin, emerge with aging and expand in neurodegeneration, linking glial crosstalk failure to the white matter pathology that often precedes overt cognitive decline (Safaiyan et al., 2021). Interferon-responsive microglia (IRM), characterized by upregulation of type I interferon-stimulated genes such as Ifitm3, Irf7, and Oasl2, represent a discrete subset that increases with amyloid burden and appears more prominent in advanced pathology and in female animals (Mathys et al., 2017; Sala Frigerio et al., 2019). Lipid-droplet-accumulating microglia (LDAM), which accumulate neutral lipids and display defective phagocytosis, elevated ROS, and a pro-inflammatory secretome, provide a mechanistic bridge between age-related metabolic dysfunction and impaired clearance (Marschallinger et al., 2020). Together with DAM and dystrophic microglia, these states are not interchangeable: they differ in their dependence on TREM2 and APOE, their interferon and lipid-handling programs, and their capacity for productive versus maladaptive communication with astrocytes.

Astrocytes have undergone a similar conceptual transition. The A1/A2 framework emerged from studies showing that microglia-derived IL-1α, TNF-α, and C1q could induce a reactive astrocyte state that lost support functions and secreted neurotoxic factors, whereas ischemia-related reactive astrocytes could express neuroprotective programs (Yun et al., 2018; Park et al., 2021). This model has been influential, but it now appears too binary. Disease-associated astrocytes in AD display diverse signatures involving inflammatory genes, lysosomal pathways, metabolic changes, proteostatic networks, and interactions with plaques (Mucke et al., 2000; Habib et al., 2020; Zhang et al., 2025). Importantly, astrocyte reactivity is not uniform across the brain. Astrocytes exhibit pronounced regional molecular and morphological heterogeneity under baseline conditions, and this diversity shapes how they respond to pathology: hippocampal, cortical, and striatal astrocytes differ in their transcriptomic identities and in the reactive programs they adopt (Endo et al., 2022; Labarta-Bajo and Allen, 2025). Such region-specific diversity may help explain why particular circuits—notably the hippocampus and deep cortical layers—are selectively vulnerable, since the local astrocytic repertoire determines the buffering capacity and complement tone available to neighboring microglia and neurons. Some may initially be protective, forming barriers or supporting clearance, before later becoming detrimental as lysosomal capacity, metabolic support, or anti-inflammatory feedback are exhausted (Blasko et al., 2000; Zhao et al., 2011; Söllvander et al., 2016).

The practical consequence of these observations is profound: glial states in AD should be interpreted as relational, not intrinsic. Microglial and astrocytic phenotypes emerge from ongoing crosstalk with each other, with neurons, with vascular elements, and with the broader metabolic and immune environment. A microglial state that is beneficial in the presence of competent astrocytic support may become pathogenic when astrocytes have already lost homeostatic functions. Likewise, an astrocytic response that is protective in the setting of restrained microglial surveillance may become neurotoxic when accompanied by excessive complement release and persistent inflammatory cytokine exposure.

This relational view helps resolve several apparent contradictions in the field. It explains why interventions that “activate” microglia can either improve or worsen pathology depending on timing; why reactive astrocytes can form beneficial barriers around plaques in one stage but amplify neurotoxicity in another; and why identical molecular markers may carry different biological meanings across disease contexts. In short, AD is not driven by fixed glial identities, but by shifting communication states.

Mapping these populations onto our three-stage framework clarifies their divergent roles. During the preclinical stage, aging-associated priming, WAM, LDAM, and low-grade interferon-responsive signatures likely dominate, reflecting metabolic and myelin stress that erodes homeostatic reserve before overt pathology (Marschallinger et al., 2020; Safaiyan et al., 2021). During the compensatory stage, TREM2/APOE-dependent DAM and plaque-associated ARM, together with barrier-forming disease-associated astrocytes, represent the organized defensive response that contains plaques and clears tau. During the maladaptive stage, the accumulation of dystrophic and terminally inflammatory microglia, LDAM with failed phagocytosis, and neurotoxic (C3^+^) disease-associated astrocytes marks the collapse of productive crosstalk, when the glial network shifts from containment to propagation. This framework predicts that the same intervention—for example, TREM2 agonism or complement inhibition—will have opposite consequences depending on which glial populations predominate at the time of treatment.

## 6 Microglia–astrocyte crosstalk in Aβ pathology: from containment to distortion

Amyloid-beta accumulation remains one of the earliest and most intensively studied events in AD. Yet plaques are not inert deposits. They are dynamic microenvironments around which multiple cell types reorganize, communicate, and compete (Wegiel et al., 2000; Adlard and Vickers, 2002; Bouvier et al., 2016). The behavior of microglia and astrocytes around Aβ plaques offers one of the clearest examples of how protective crosstalk can gradually become maladaptive.

### 6.1 Early containment: the adaptive logic of glial convergence

The association between microglia and Aβ plaques has been recognized since the early 1990s (Wisniewski et al., 1989, 1991). In human AD brains and mouse models, microglia cluster tightly around dense-core plaques and also appear near diffuse deposits (Haga et al., 1989; Akiyama et al., 1999). *In vivo* imaging studies show that plaque growth can be rapid and that microglial processes extend toward newly forming plaques shortly after their appearance (Bacskai and Hyman, 2002; Bolmont et al., 2008). Their coverage of the amyloid surface is not incidental. It contributes to plaque compaction, restricts plaque expansion, and may reduce exposure of surrounding neurites to highly toxic Aβ species (Spangenberg et al., 2019; Casali et al., 2020).

Microglia interact with diverse Aβ conformations—including soluble oligomers, protofibrils, and insoluble fibrils—through a broad range of receptors such as CD14, CD36, CD47, integrins, SCARA1, and TLRs (Mizuno, 2012). These interactions promote uptake, degradation, and the release of cytokines, ROS, and complement components. The consequence is ambivalent. On the one hand, early microglial activation enhances Aβ clearance and limits deposition (Bolmont et al., 2008). On the other hand, persistent activation can impair phagocytic efficiency and sustain inflammatory amplification.

Astrocytes are also recruited to plaque-associated environments, although their logic of recruitment appears different. Whereas microglia seem to be directly attracted by Aβ itself (Dickson et al., 1988), astrocytes may respond more strongly to plaque-associated neuritic injury and local tissue disruption (Adlard and Vickers, 2002; Simpson et al., 2010). Reactive astrocytes accumulate around plaques, often forming dense hypertrophic barriers that appear to isolate the lesion from healthier tissue (Sofroniew and Vinters, 2010; Perez-Nievas and Serrano-Pozo, 2018). Experimental reduction of astrocytic reactivity by deleting GFAP and vimentin impairs astrocyte–plaque contact and increases dystrophic neurites near plaques, supporting the idea that astrocyte reactivity can be structurally protective (Kraft et al., 2013; Gomez-Arboledas et al., 2018; Sanchez-Mico et al., 2021).

This early convergence of microglia and astrocytes around Aβ deposits is best understood as a form of compensatory tissue organization. It is not just “inflammation”; it is an attempt to spatially contain a persistent source of proteotoxic stress.

### 6.2 Plaque microenvironments are communication hubs, not passive lesions

The interaction between glia and plaques is not reducible to simple recruitment. Three-dimensional imaging has revealed reactive glial nets (RGNs), in which microglia occupy the plaque-proximal core and astrocytes form a more peripheral, intertwined layer (Wegiel et al., 2000; Bouvier et al., 2016). These structures differ depending on plaque morphology. Fibrillar plaques are associated with deeper penetration of both microglial and astrocytic processes and with greater neuronal pathology than dense-core plaques (Adlard and Vickers, 2002). This suggests that glial architecture around plaques is itself a meaningful biological variable, potentially influencing the degree of local neurotoxicity.

Microglia–astrocyte communication is crucial in determining this architecture. Astrocytic signals such as APOE, Clusterin, and CD44-linked pathways interact with the TREM2–DAP12 axis in microglia, shaping disease-associated microglial programs near plaques (Wang C. et al., 2021). Astrocyte-specific Apoe deletion reduces DAM-associated markers, indicating that astrocytes actively instruct microglial phenotypic transitions in plaque-rich environments (Wang C. et al., 2021). Conversely, microglia-derived cues influence astrocytic arrangement and reactivity. Plexin-B1, expressed in reactive astrocytes, has emerged as a regulator of astrocyte and microglia positioning around plaques (Wang C. et al., 2021; Huang et al., 2024). Deletion of Plexin-B1 in AD models reduces plaque burden, promotes plaque compaction, limits neuroinflammation, and improves cognition, apparently by allowing tighter glial coverage and more efficient plaque isolation.

The Sema4D–Plexin axis may be one route through which microglia control astrocytic spacing (Clark et al., 2021; Evans et al., 2022). Although most of this evidence comes from related models rather than exclusively AD, it raises an important possibility: microglia may not only trigger astrocyte activation, but also shape the geometry of astrocytic responses. Plaque-associated pathology, then, emerges from a spatially organized crosstalk network rather than from isolated cellular reactions.

### 6.3 Aβ clearance: a cooperative function that fails with time

Both microglia and astrocytes can clear Aβ, although through different mechanisms and with different limitations. Microglia are specialized phagocytes, but their efficiency decreases with chronic exposure, inflammatory overload, and metabolic stress (Spangenberg et al., 2019; Casali et al., 2020; Kiani Shabestari et al., 2022). Astrocytes can internalize Aβ, express Aβ-degrading enzymes such as neprilysin and insulin-degrading enzyme, and secrete matrix metalloproteinases that promote extracellular Aβ degradation (Wyss-Coray et al., 2003; Koistinaho et al., 2004; Liu et al., 2016). At least in early disease, this division of labor may be advantageous: microglia specialize in direct uptake and plaque handling, whereas astrocytes provide structural buffering, extracellular degradation, and metabolic stabilization.

Yet this cooperative system is fragile. In some conditions, astrocytes can themselves become Aβ-producing cells under the influence of TGF-β1, IFN-γ/TNF-α, or IFN-γ/IL-1 signaling (Blasko et al., 2000; Lesné et al., 2003; Zhao et al., 2011). Astrocytes that engulf large amounts of partially digested Aβ protofibrils may lose degradative capacity and promote neuronal apoptosis (Söllvander et al., 2016). Age-related lysosomal dysfunction further limits their clearance competence (Wolfe et al., 2013; Xiao et al., 2014; Lee et al., 2022; Yang et al., 2024). Once astrocytic support is impaired, microglia face a harsher microenvironment with greater inflammatory and oxidative load, which may further reduce their ability to sustain effective phagocytosis.

**FIGURE 3:**
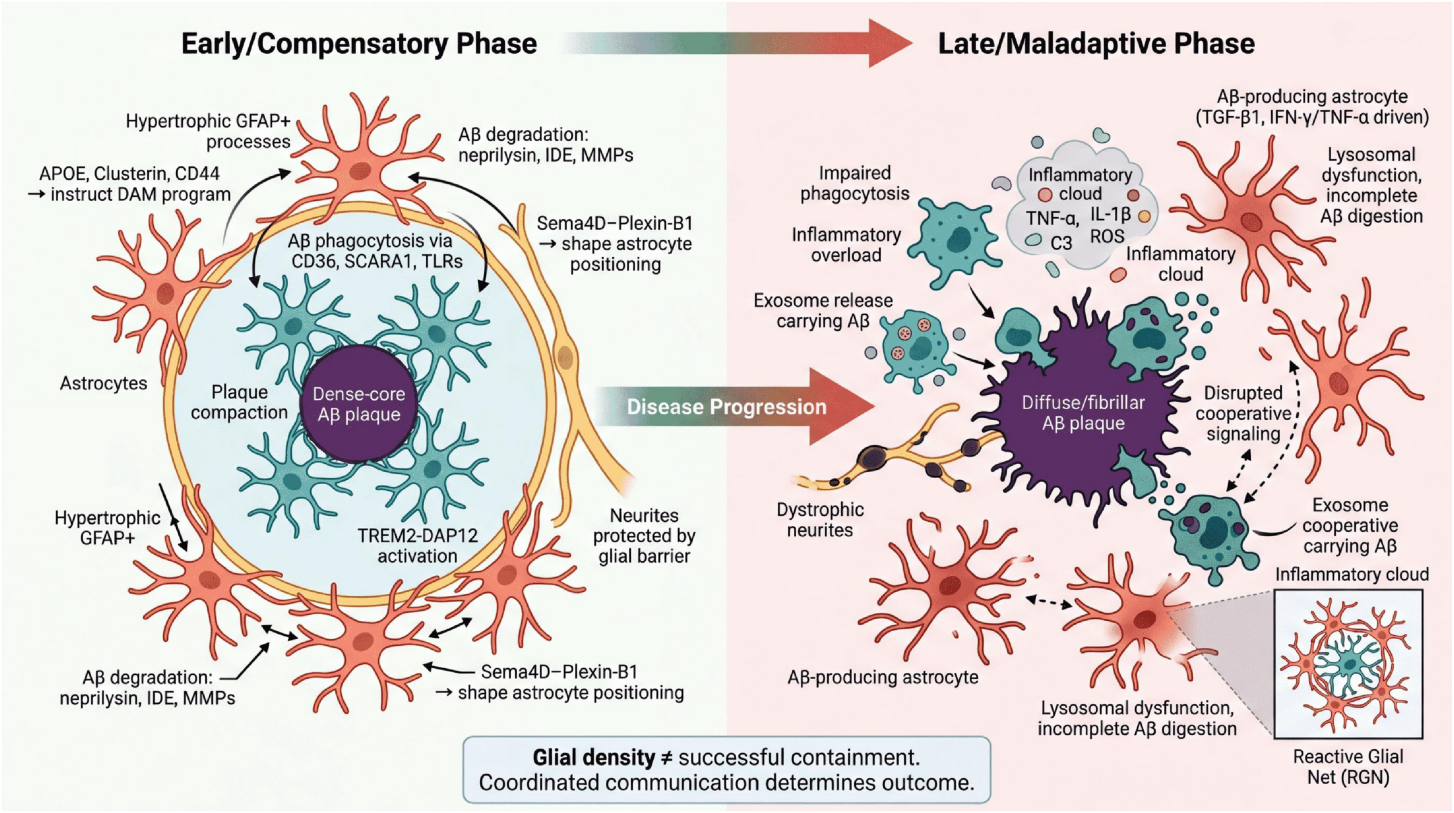
Glial crosstalk around amyloid-beta (Aβ) plaques transitions from compensatory containment to maladaptive distortion. In the early phase (left), microglia compact dense-core plaques via CD36/SCARA1/TLRs and TREM2–DAP12 signaling, while hypertrophic GFAP^+^ astrocytes form protective barriers and degrade Aβ through neprilysin, IDE, and MMPs. Astrocytic APOE/Clusterin/CD44 instruct the microglial DAM program; Sema4D–Plexin-B1 shapes astrocyte positioning. In the late phase (right), plaques become diffuse/fibrillar, microglia show impaired phagocytosis and release Aβ-carrying exosomes, while astrocytes become Aβ-producing cells with lysosomal dysfunction. Inflammatory mediators TNF-α,IL-1ß,C3,ROS accumulate, and cooperative signaling collapses. Inset: reactive glial net (RGN). Glial density does not equal successful containment—coordinated communication determines outcome.

This failure of cooperation may explain why glial accumulation around plaques does not necessarily imply plaque resolution. In fact, increasing glial density can coexist with worsening pathology if the underlying communication has already become distorted. A plaque surrounded by glia is not necessarily a sign of successful containment; it may indicate a stalled or dysregulated attempt at containment (Figure 3).

### 6.4 Conflict point: more glial activation does not always mean worse disease—and less glial activation does not always mean better disease

A useful but uncomfortable conclusion emerges here: the field has often treated glial activation as if it were inherently harmful, when in reality insufficient or disorganized glial engagement can be equally damaging. Microglial depletion studies show that removing microglia from AD models can worsen plaque architecture, increase amyloid angiopathy, promote calcification and hemorrhage, and shorten survival (Spangenberg et al., 2019; Casali et al., 2020). Likewise, impairing astrocyte reactivity can increase neuritic dystrophy around plaques (Kraft et al., 2013; Gomez-Arboledas et al., 2018; Sanchez-Mico et al., 2021). These findings directly challenge the notion that the goal should be generalized suppression of glial responses.

The relevant question is therefore not whether glia are activated, but whether their communication remains functionally coordinated. A highly reactive but well-organized glial network may still restrict injury. A moderately reactive but disorganized network may fail catastrophically. This distinction may help explain the mixed outcomes of anti-inflammatory interventions in AD and should be given greater weight in future therapeutic design.

## 7 Microglia–astrocyte crosstalk in tau pathology: clearance, propagation, and inflammatory escalation

If Aβ deposits are prominent extracellular focal points of glial interaction, tau pathology exposes a different dimension of glial crosstalk: the tension between degradative defense and network-level propagation. Tau hyperphosphorylation, misfolding, and aggregation correlate closely with neuronal dysfunction and clinical decline (Luo et al., 2015; Maphis et al., 2015; Bolós et al., 2016; Clayton et al., 2021). Although tau is often framed as a neuronal pathology, growing evidence indicates that microglia and astrocytes profoundly shape its spread, toxicity, and tissue consequences.

### 7.1 Microglia and astrocytes as tau-clearing cells

Microglia can internalize and degrade pathological tau species *in vivo* and *in vitro* (Brelstaff et al., 2018; Hopp et al., 2018). Hyperphosphorylated tau isolated from AD patients or P301S mice can be taken up by microglia and processed through intracellular degradative pathways (Luo et al., 2015). Microglia also colocalize with NFTs in postmortem AD tissue (Bolós et al., 2016), suggesting active engagement with tau-bearing structures. Astrocytes may participate more indirectly, but they too can internalize tau, particularly under conditions of extracellular tau accumulation or advanced tauopathy (Ferrer et al., 2014; Kahlson and Colodner, 2015; Leyns and Holtzman, 2017). In principle, therefore, glial cells provide a first line of defense against extracellular and trans-synaptically released tau.

Astrocyte-derived IL-3 appears to participate in this protective logic by binding IL-3Rα on microglia, altering microglial transcriptional and morphological states, and enhancing their clustering around tau deposits (Luo et al., 2015; Taddei et al., 2023). This example is especially instructive because it reverses a common assumption: astrocytes are not merely recipients of microglial inflammatory signals but can actively direct microglial anti-tau behavior. Such findings argue for a more symmetrical model of glial crosstalk in which either cell type can become the upstream regulator depending on the pathological context.

### 7.2 When clearance becomes propagation

The protective function of glia in tauopathy is limited by degradative capacity. Microglia that over-engulf tau may fail to fully degrade it, enter a stressed or degenerative state, and subsequently release tau-containing material, thereby facilitating tau propagation (Sanchez-Mejias et al., 2016; Hopp et al., 2018). Exosomes are one probable vehicle of this spread (Goetzl et al., 2016). In rodent models, microglia have been shown to promote the dissemination of tau pathology through exosomal secretion, and depletion of microglia markedly reduces tau propagation (Goetzl et al., 2016). This is one of the clearest examples of how a protective cellular function—phagocytosis—can become pathogenic when not coupled to efficient degradation.

The same paradox likely applies to astrocytes. Astrocytes that internalize tau may initially buffer extracellular toxicity but later contribute to local inflammation or altered support functions if their proteostatic machinery becomes overwhelmed (Ikeda et al., 1992; Amro et al., 2021; Smith et al., 2022). In advanced disease, thorn-shaped astrocytes and aging-related tau astrogliopathy indicate that astrocytes are not passive spectators in tau-driven neurodegeneration (Ferrer et al., 2014; Kahlson and Colodner, 2015). These changes further reinforce the idea that glial phenotypes are not fixed categories, but outcomes of ongoing crosstalk under conditions of variable proteostatic stress.

It should be emphasized that the causal steps in this clearance-to-propagation transition—microglial engulfment, failed degradation, and re-release of seeding-competent tau—have been established largely in transgenic rodents and cell culture; their quantitative contribution to tau spreading in the human brain remains inferred rather than directly demonstrated.

### 7.3 Cytokine and complement circuits amplify tau pathology

A second major contribution of glial crosstalk to tau pathology lies in inflammation-induced tau modification. Pro-inflammatory mediators including TNF-α, IL-1β, IL-6, and complement components can promote tau phosphorylation and exacerbate neuronal dysfunction (Bhaskar et al., 2010; Kitazawa et al., 2011; Kahlson and Colodner, 2015). These mediators are generated largely by activated microglia and reactive astrocytes, often in mutually reinforcing loops. Reduced CX3CL1–CX3CR1 signaling in AD brains and tauopathy models has been associated with increased microglial activation and elevated tau phosphorylation. Genetic changes in microglia-related pathways including CSF1R, APOE, and TREM2 further influence tau-driven neurodegeneration (Shi et al., 2017, 2019; Leyns et al., 2019).

**FIGURE 4:**
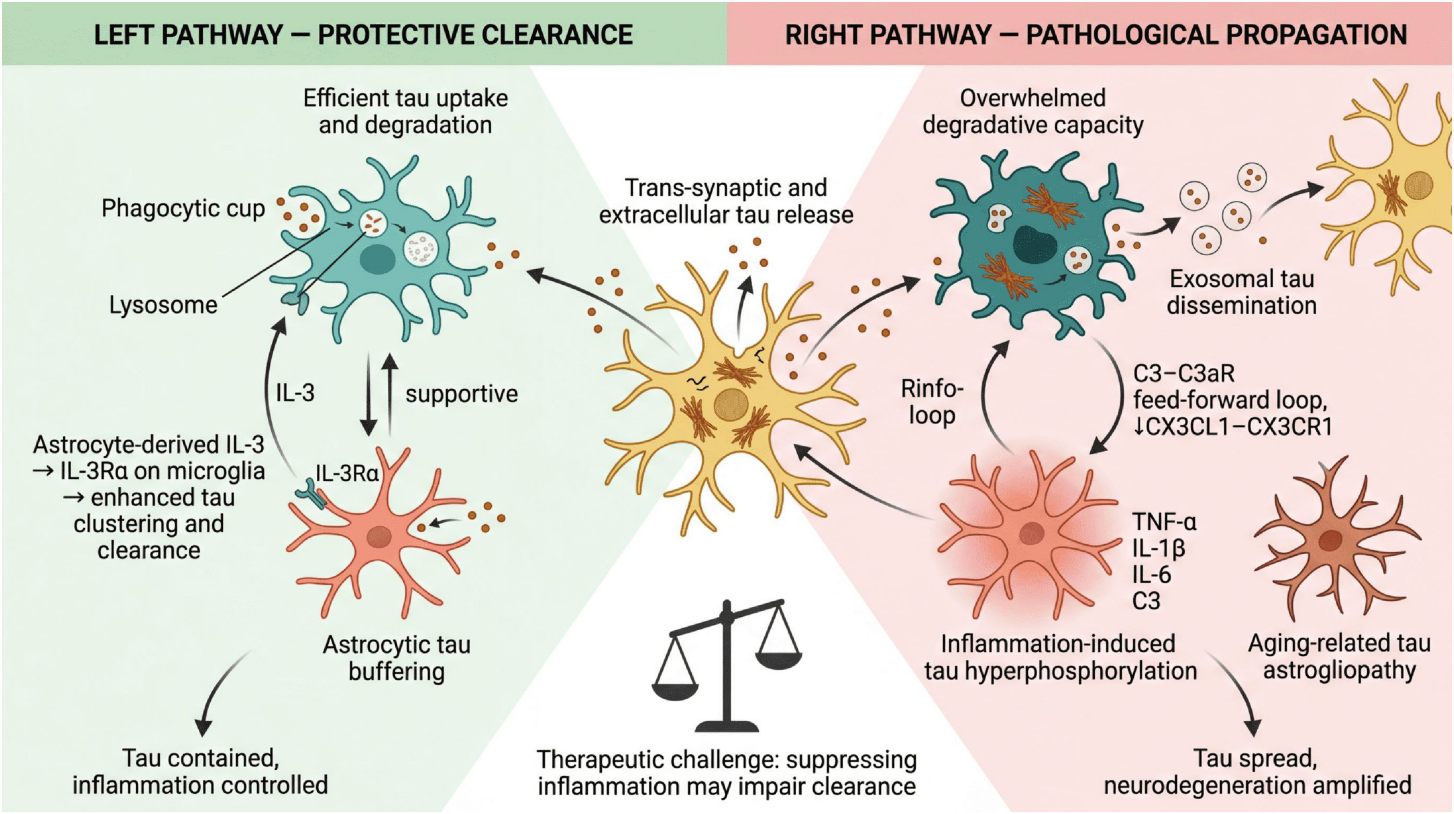
Microglia–astrocyte crosstalk mediates both tau clearance and tau propagation. A central tau-bearing neuron releases tau seeds trans-synaptically. In the protective pathway (left), microglia efficiently uptake and degrade tau via phagocytic-lysosomal processing, supported by astrocyte-derived interleukin-3 (IL-3) binding microglial IL-3Rα to enhance tau clustering and clearance; astrocytes also buffer extracellular tau. In the pathological pathway (right), overwhelmed microglia release tau-containing exosomes, driving dissemination. Reactive astrocytes and microglia form complement component 3 (C3)–complement C3a receptor (C3aR) feed-forward inflammatory loops (↓CX3CL1–CX3CR1), releasing tumor necrosis factor alpha (TNF-α), interleukin-1 beta (IL-1β), interleukin-6 (IL-6), and C3, promoting inflammation-induced tau hyperphosphorylation and aging-related tau astrogliopathy. Balance icon: suppressing inflammation may impair clearance.

Complement signaling, especially via the C3–C3aR axis, has emerged as a crucial link between microglia–astrocyte communication and tau pathology (Lian et al., 2015). C3 produced by reactive astrocytes and related glial interactions can activate C3aR on microglia, thereby modulating microglial responses, synaptic function, and inflammatory tone. Gene knockout studies of C3aR reduce neuroinflammation, synaptic loss, and tau pathology in tauopathy models (Lian et al., 2015). Downstream effectors such as STAT3 also participate in this harmful axis (Leyns and Holtzman, 2017; Litvinchuk et al., 2018; Taddei et al., 2022). Thus, complement acts here not merely as a terminal effector of innate immunity, but as a language of crosstalk through which astrocytes and microglia co-regulate tau-associated injury (Figure 4).

### 7.4 Conflict point: inhibiting glial tau responses may reduce inflammation but impair clearance

Tau pathology crystallizes one of the most important therapeutic dilemmas in AD. Because activated microglia and reactive astrocytes can exacerbate tau phosphorylation and spread, there is an obvious rationale for suppressing glial inflammation. Yet those same cells are also essential for tau recognition, uptake, and containment. A treatment that broadly reduces glial activation may diminish toxic cytokines while simultaneously weakening tau clearance. Conversely, a therapy that enhances phagocytosis without supporting degradative competence may accelerate tau dissemination.

This is not a minor methodological complication; it is a conceptual warning. There is no universally beneficial direction of glial manipulation in tauopathy. What matters is whether the intervention selectively promotes degradative competence, limits inflammatory spillover, and preserves tissue support functions. A therapy that does only one of these may fail—or even backfire.

## 8 Neuroinflammation as a communication disorder rather than a cell-autonomous response

Neuroinflammation is a defining feature of AD, but it is often described as though it were simply the sum of activated microglia plus reactive astrocytes (Calsolaro and Edison, 2016; Kaur et al., 2019). This view is too crude. Neuroinflammation in AD is better understood as a communication disorder in which microglia and astrocytes exchange signals that progressively detach from homeostatic regulation and become self-reinforcing.

To make this claim testable rather than merely descriptive, we define our two central terms operationally. We define a maladaptive signaling loop as a reciprocal glial signaling circuit that satisfies three empirically checkable criteria: (i) self-sustainment—the circuit persists, in experimental models, after removal or neutralization of the initiating stimulus (e.g., after Aβ lowering); (ii) node-dependence—interruption at a single molecular node (e.g., C3aR, IL-1α, or C1q) measurably reduces downstream pathology; and (iii) net functional cost—the circuit is associated with a decline in a quantifiable tissue-function readout, principally synapse density. We define communication collapse as the point at which glial output ceases to track, or respond correctively to, the local pathological burden—operationally, the decoupling of glial reactivity from glial clearance function, such that reactivity markers continue to rise while measurable clearance of Aβ, tau, or debris falls.

### 8.1 Microglia usually initiate; astrocytes determine spread and persistence

Microglia are more broadly equipped than astrocytes for primary pathogen recognition, expressing a wider array of TLRs and related innate immune sensors (Bsibsi et al., 2002; Kielian, 2006; Rodríguez-Gómez et al., 2020). Astrocytes, with their more limited TLR repertoire, often depend on signals from activated microglia to enter robust inflammatory states (Li et al., 2021; Garland et al., 2022). This asymmetry means that microglia frequently act as first responders in CNS inflammation. In cultured human cells, astrocytes can respond strongly to IL-1 released from activated microglia even when they respond poorly to LPS itself (Lee et al., 1993). Similar logic likely applies in AD, where Aβ and DAMPs activate microglia, which then transmit inflammatory cues to astrocytes.

The canonical example is the microglia-derived trio of IL-1α, TNF-α, and C1q, which induces neurotoxic reactive astrocytes (Yun et al., 2018; Park et al., 2021; Li et al., 2022). These astrocytes lose multiple homeostatic functions—including support for neuronal survival, synaptogenesis, and phagocytosis—and can secrete factors harmful to neurons and oligodendrocytes (Liddelow et al., 2017). Elevated levels of these cytokines and increased A1-like astrocyte signatures have been observed in AD-relevant contexts, and interventions such as the GLP-1R agonist NLY01 can attenuate this axis and preserve neuronal function (Yun et al., 2018; Park et al., 2021). This strongly supports the idea that microglial inflammatory output is not merely damaging in itself; it also reprograms astrocytes into secondary amplifiers of pathology.

### 8.2 Astrocytes can also restrain microglial inflammation

The relationship, however, is bidirectional. Astrocytes can produce anti-inflammatory or regulatory mediators that reshape microglial behavior. Astrocyte-derived IL-10 and TGF-β signaling can suppress M1-like microglial activation and promote a more reparative environment (Bernaus et al., 2020; O’Neil et al., 2022). TGF-β has even been shown to protect synapses against Aβ oligomer toxicity (Diniz et al., 2017), although its signaling may be impaired in AD brains (Lee et al., 2006; Lian et al., 2016). Astrocytic ORM2 can inhibit microglial migration and activation through blockade of microglial CCR5, producing anti-inflammatory effects. Astrocyte-derived BDNF and GDNF also modulate microglial activation and can inhibit pro-inflammatory outputs (Rocha et al., 2012; Lai et al., 2018).

These findings carry an underappreciated implication: astrocytes are not simply converted by microglia from a resting into a reactive state. They also provide negative feedback. Neuroinflammation becomes chronic in part because this regulatory astrocytic brake weakens with aging and disease progression.

### 8.3 Extracellular vesicles and paracrine loops widen the inflammatory field

In addition to classical cytokines, EVs have emerged as major mediators of glial inflammatory communication (Goetzl et al., 2016; Paolicelli et al., 2019; d’Errico et al., 2022). ATP released by neighboring astrocytes can stimulate microglial EV shedding, while microglial EVs can carry IL-1β and other inflammatory cargos that modulate astrocytes and spread local signals into broader tissue domains (Drago et al., 2017). Activated microglia-derived exosomes can also transport proteins associated with adhesion, extracellular matrix remodeling, metabolism, and autophagy–lysosomal pathways, thereby reshaping astrocyte behavior (Drago et al., 2017). Because EVs can carry aggregated Aβ and tau as well, they sit at the intersection of inflammatory and proteopathic signaling (Goetzl et al., 2016; Paolicelli et al., 2019).

This broadening of the communication field is crucial for understanding how localized pathology becomes network pathology. Once inflammatory signals are vesicle-mediated, the pathological conversation is no longer limited to adjacent cells. Entire microdomains of the brain can become transcriptionally and metabolically reprogrammed.

### 8.4 Conflict point: anti-inflammatory therapy fails when it targets inflammatory molecules but not the communication architecture

A large part of the disappointment surrounding anti-inflammatory therapy in AD may reflect a mismatch between target choice and disease organization. Many approaches attempt to reduce specific cytokines or inflammatory pathways. Yet neuroinflammation in AD is not merely the presence of inflammatory molecules. It is the persistent, multi-channel exchange of signals among glial cells, neurons, and vascular compartments. Blocking a single mediator may dampen one node of this network while leaving its architecture intact.

This may be why broad anti-inflammatory strategies often produce underwhelming outcomes despite abundant evidence of inflammatory activation. Unless the therapy restores the balance between initiating, amplifying, and restraining arms of glial communication, the system may simply reroute through parallel pathways. In that sense, AD neuroinflammation behaves less like a linear cascade and more like a robust pathological network.

## 9 Complement, chemokines, and direct signaling routes: the molecular language of glial crosstalk

The communication between microglia and astrocytes in AD relies on a dense repertoire of soluble and contact-dependent signals. Among them, complement and chemokine systems are especially important because they connect immune signaling with phagocytosis, synapse remodeling, and plaque/tau handling.

### 9.1 Complement as both physiological code and pathological amplifier

The complement cascade plays essential roles in development and tissue maintenance, where it helps identify material destined for clearance (Fonseca et al., 2017; Datta et al., 2020). In AD, however, this system is repurposed and amplified. Aβ exposure activates NF-κB signaling in astrocytes, increasing C3 production (Lian et al., 2015). C3 then engages C3aR on microglia and neurons, influencing phagocytosis, cognition, and inflammatory tone (Lian et al., 2015). Short-term C3 exposure can transiently enhance microglial Aβ uptake, but prolonged exposure reduces it, revealing a time-dependent inversion of effect (Lian et al., 2015). This is a striking example of how molecules that initially support adaptive clearance can later promote dysfunction.

C3aR is not the only relevant receptor. CR3, expressed by microglia, recognizes complement-tagged synapses and contributes to synaptic pruning (Shi et al., 2015; Anderson et al., 2019). C1q and C3 accumulation at synapses are increased in aging and AD, correlating with excessive synaptic phagocytosis and cognitive decline (Dejanovic et al., 2018; Wu et al., 2019). Meanwhile, astrocytic C3 and microglial C3aR together constitute a feed-forward loop that worsens Aβ dynamics and neuropathology (Lian et al., 2015). Thus, complement is best understood as a dual-use signaling system—necessary for physiological surveillance, but harmful when chronic, mislocalized, or insufficiently opposed. These time- and dose-dependent effects of the C3–C3aR axis derive from experimental systems; in human AD, C3 and C3aR are consistently found elevated and spatially associated with synapse loss, but such data remain correlative.

### 9.2 Chemokines coordinate glial migration and mutual recruitment

Chemokine signaling adds another layer of bidirectional control. Astrocytes release CCL2, CXCL1, CXCL10, and CXCL12, while microglia express matching receptors and respond with migration or activation (Andjelkovic et al., 2002; Conductier et al., 2010). Although there is some controversy about chemotactic responses in astrocytes, the dominant evidence suggests that microglia are more chemotactically responsive and therefore more likely to be recruited rapidly into injury fields (Peterson et al., 1997; Conductier et al., 2010). Elevated CCL2 levels correlate with memory impairment and are increased in the plasma of MCI and AD patients (Galimberti et al., 2006; Bettcher et al., 2016, 2019; Lee et al., 2018). CCL4 is widely expressed by reactive astrocytes in AD brains (Xia et al., 1998), again suggesting that astrocytes contribute actively to the chemotactic environment that shapes microglial distribution.

Such chemokine systems not only mobilize glia; they influence the geometry of glial contact with plaques, synapses, and vascular elements. The spatial organization of pathology is therefore partly a chemokine-organized phenomenon.

### 9.3 Paracrine regulators with therapeutic implications

Beyond complement and chemokines, several molecules illustrate the specificity of glial crosstalk. Astrocytic ORM2 restrains microglial activation through CCR5-related mechanisms (Jo et al., 2017). PAI-1, produced predominantly by astrocytes under inflammatory conditions, regulates microglial migration and phagocytic activity via LRP-1/JAK/STAT1 and TLR2/6-related mechanisms (Liu R.-M. et al., 2011; Akhter et al., 2018). Elevated PAI-1 in AD patients and the beneficial effects of PAI-1 inhibition in APP/PS1 mice suggest that microglia–astrocyte crosstalk can be therapeutically manipulated through non-canonical immune pathways (Liu R.-M. et al., 2011; Akhter et al., 2018). Astrocyte-derived GDNF and related trophic factors likewise influence microglial activation states and may slow neurodegeneration (Rocha et al., 2012; Lai et al., 2018).

These examples reinforce the point that glial communication is not a generic inflammatory blur. It is a structured molecular language. The challenge is to identify which elements of this language are adaptive, which become distorted during disease, and which can be modulated without disabling essential tissue functions.

## 10 Synapse loss as the major functional output of maladaptive glial crosstalk

Among all pathological features of AD, synapse loss correlates most closely with cognitive decline (Südhof, 2017; Griffiths and Grant, 2023; Wen et al., 2024). This glial contribution operates alongside, not instead of, well-established synaptic and network mechanisms—including Aβ oligomer– and tau-driven synaptic depression, impaired long-term potentiation, and circuit hyperexcitability (Palop and Mucke, 2016; Busche and Hyman, 2020). We propose that maladaptive complement- and phagocytosis-mediated synapse elimination is one route by which these neuronal processes are amplified into net synaptic loss, providing a point of convergence rather than an alternative explanation. A growing body of experimental evidence indicates that this synaptic pathology is not simply caused by neuronal toxicity from Aβ and tau. Rather, in animal models it appears to be actively executed—and often over-executed—by microglia and astrocytes through a reactivation of developmental pruning programs under pathological conditions. In humans, complement-mediated synaptic tagging and glial engulfment of synaptic material have been documented histologically (Taddei et al., 2023), but the causal sequence is inferred primarily from model systems.

### 10.1 Physiological pruning becomes pathological engulfment

During development and in adult circuit remodeling, microglia and astrocytes remove weak or unnecessary synapses through coordinated engulfment pathways (Fonseca et al., 2017; Datta et al., 2020; Cornell et al., 2022). Complement components C1q and C3 label synapses for microglial recognition by CR3 (Fonseca et al., 2017; Anderson et al., 2019). Astrocytes use MEGF10 and MERTK to phagocytose synaptic structures (Chung et al., 2013). These pathways are essential for normal circuit refinement. In AD, however, they are reactivated inappropriately.

Excessive complement deposition at synapses is seen in aged brains and AD models (Dejanovic et al., 2018; Wu et al., 2019). Microglia then engulf complement-tagged synapses beyond what is needed for adaptive remodeling. “Eat Me” signals such as phosphatidylserine (PS), recognized by TREM2 and related receptors, further promote synaptic pruning (Sapar et al., 2018; Zhang et al., 2018; Nemes-Baran et al., 2020). At the same time, protective “Don’t Eat Me” signals such as CD47–SIRPα weaken, removing critical inhibitory brakes on microglial phagocytosis (Oldenborg et al., 2001; Lehrman et al., 2018; Ding et al., 2021). The result is not random synapse loss, but a pathological extension of normal clearance logic into functionally intact neural circuits.

### 10.2 Astrocytes are not bystanders in synapse elimination

Astrocytes have often been overshadowed by microglia in discussions of synaptic pruning, yet their role in AD is increasingly evident. In APP/PS1 mice, astrocytes display increased contacts with synapses and signs of enhanced phagolysosomal activity (St-Pierre et al., 2023). They can envelop, phagocytose, and degrade damaged neurites near plaques, although the proportion of affected neurites they successfully clear remains limited (Gomez-Arboledas et al., 2018; Sanchez-Mico et al., 2021). Importantly, Aβ can impair astrocytic phagocytic function, and reduced expression of MERTK and MEGF10 in AD may further compromise astrocyte-mediated synaptic clearance (Hulshof et al., 2022). This suggests a double failure: astrocytes become more engaged with damaged synapses but less competent at resolving them efficiently.

That dysfunction matters because astrocytes help set the conditions under which microglial pruning occurs. Through complement release, glutamate transport, cytokine signaling, and local metabolic buffering, astrocytes influence whether microglial engulfment remains selective or becomes excessive.

### 10.3 Astrocyte–microglia communication governs synaptic vulnerability

One particularly important example is the astrocytic C3/microglial C3aR axis. Excessive Aβ can activate astrocytic NF-κB signaling and drive C3 secretion (Lian et al., 2015). C3 engagement of microglial C3aR alters dendritic spine structure, modulates phagocytic behavior, and contributes to cognitive dysfunction (Lian et al., 2015, 2016). Metabotropic glutamate receptor signaling is also increased in AD-related contexts and associates with enhanced synaptic C1q and microglial pruning (Bie et al., 2019). Restoring astrocytic GLT-1 reduces C1q accumulation, decreases microglial synaptic phagocytosis, and ameliorates synaptic and cognitive deficits in Aβ-exposed models (Wu et al., 2020). These data are highly instructive because they provide causal, loss- and gain-of-function evidence—albeit in rodent models—that a nominally “neuronal” phenomenon—synaptic dysfunction—can be corrected by manipulating astrocyte–microglia communication rather than neuronal receptors alone. Whether analogous causal relationships hold in the human brain remains to be established.

**FIGURE 5:**
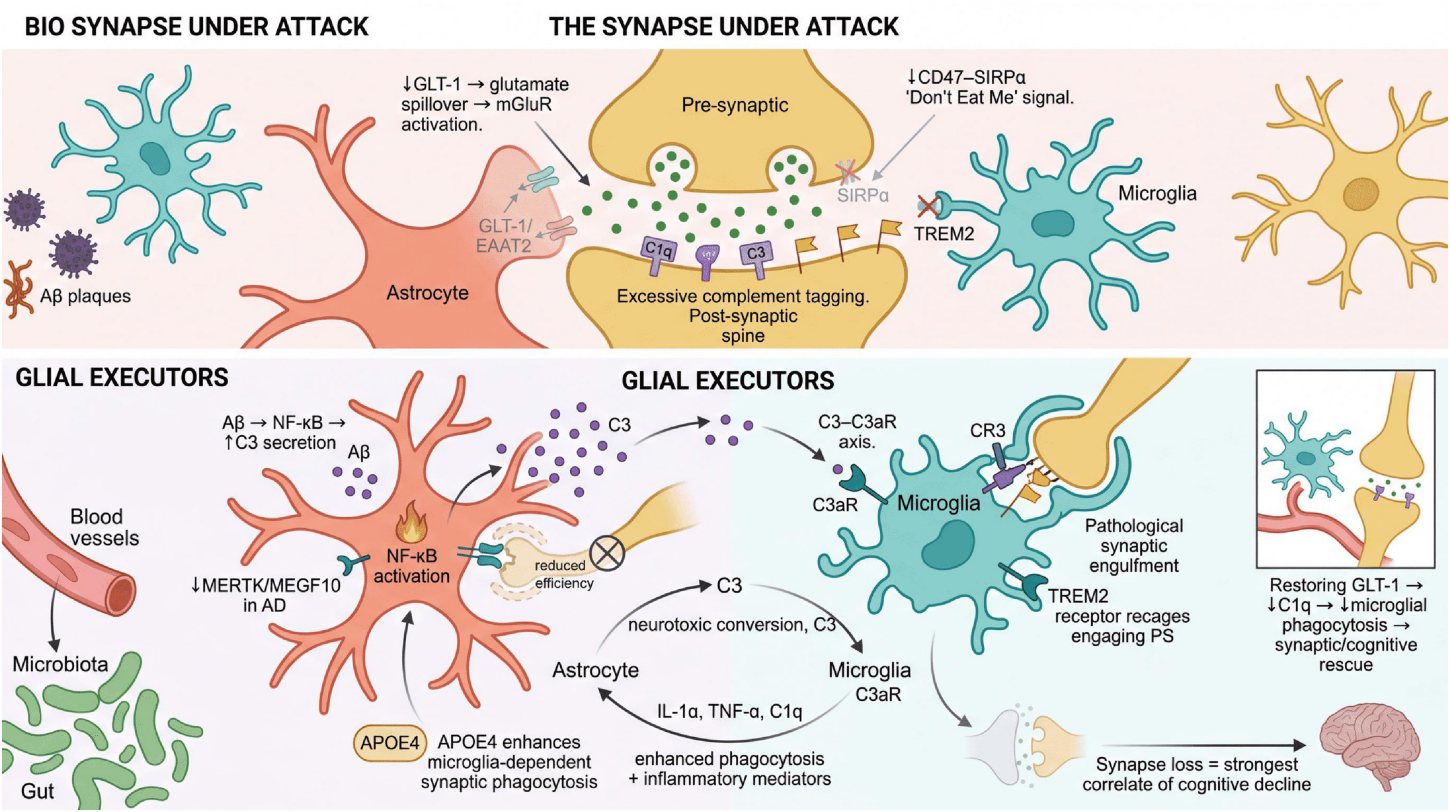
Maladaptive microglia–astrocyte communication drives pathological synapse elimination. (Top) Reduced astrocytic glutamate transporter 1 (GLT-1) causes glutamate spillover and mGluR activation; excessive complement component 1q (C1q)/complement component 3 (C3) tags accumulate on post-synaptic spines while protective cluster of differentiation 47 (CD47)–signal regulatory protein alpha (SIRPα) “Don’t Eat Me” signals weaken. Triggering receptor expressed on myeloid cells 2 (TREM2) engages phosphatidylserine on vulnerable synapses. (Bottom) Amyloid-beta (Aβ) activates astrocytic nuclear factor kappa-light-chain-enhancer of activated B cells (NF-κB), driving C3 secretion; C3– complement C3a receptor (C3aR) signaling enhances microglial phagocytic engulfment via CR3 and TREM2. Astrocytic MER proto-oncogene tyrosine kinase (MERTK)/multiple EGF-like domains 10 (MEGF10) efficiency declines in AD. A feed-forward loop (IL-1α/TNF-α/C1q → neurotoxic astrocytic conversion → more C3) sustains synapse elimination. Apolipoprotein E4 (APOE4) exacerbates microglia-dependent synaptic phagocytosis. (Inset) Restoring glutamate transporter 1 (GLT-1) reduces C1q and rescues synaptic/cognitive function. Synapse loss is the strongest correlate of cognitive decline.

APOE-related pathways add another layer. Astrocytic APOE4 enhances microglia-dependent synaptic phagocytosis in tauopathy contexts, and reducing astrocytic APOE4 expression can mitigate this process (Wang C. et al., 2021; Huang et al., 2024). IL-33, derived in part from astrocytic and related glial programs, promotes microglial synaptic engulfment during development but can also rescue Aβ-induced cognitive and synaptic deficits in APP/PS1 mice (Fu et al., 2016; Vainchtein et al., 2018). These seemingly contradictory observations again emphasize that the biological significance of synaptic phagocytosis depends on context, timing, and selectivity (Figure 5).

### 10.4 Conflict point: synaptic pruning is not pathological by definition—its pathological meaning depends on discrimination

The tendency to equate synaptic pruning with disease is understandable but misleading. Synapse elimination is essential for learning, memory updating, and circuit maintenance. The pathology in AD is not that glia prune synapses; it is that they lose the ability to distinguish which synapses should be pruned. This loss of discrimination likely results from the convergence of inflammatory priming, complement overactivation, altered neuronal activity, impaired astrocytic buffering, and the collapse of “Don’t Eat Me” safeguards.

This distinction is not semantic. Therapies aimed at globally suppressing phagocytic pathways risk freezing both harmful and beneficial pruning. The future lies instead in restoring selectivity—re-establishing the criteria by which glia decide what deserves elimination and what must be preserved.

## 11 The tryptophan–microbiota–AHR axis as a candidate upstream tuner of microglia–astrocyte crosstalk

Among the many potential modulators of glial communication, the Trp–microbiota–AHR axis is one candidate worth considering, though we emphasize at the outset that its role in AD is hypothetical and reconstructed almost entirely from non-AD experimental systems. It could, in principle, connect peripheral metabolism, gut microbial ecology, systemic immunity, and CNS inflammatory programs (Jin et al., 2014; Lee et al., 2017; Yusufu et al., 2021). This axis offers a way to understand how seemingly distant biological systems—diet, bacterial composition, intestinal homeostasis, and brain inflammation—become mechanistically linked in AD.

### 11.1 Trp metabolism links immunity, microbiota, and brain signaling

Tryptophan is not merely a precursor of serotonin. It is a metabolic hub that influences immunity, complement regulation, and multiple signaling systems (Keszthelyi et al., 2013; Gheorghe et al., 2019). Gut microbiota strongly shape Trp metabolism by converting dietary Trp into indole and numerous derivatives, including indole-3-aldehyde (I3A), indole-3-propionic acid (IPA), tryptamine, indoleacetic acid, indoleacrylic acid, indoleethanol, and indolelactic acid (Li and Young, 2013; Jin et al., 2014; Roager and Licht, 2018). Several of these metabolites function as AHR ligands. Microbial Trp metabolism therefore becomes an endocrine-like signaling system with the potential to affect distant tissues, including the brain (Mu et al., 2018).

The host kynurenine pathway provides an additional source of endogenous AHR ligands, notably kynurenine (KYN) and kynurenic acid (KYNA) (Lee et al., 2017). Dietary Trp availability influences not only peripheral immunity and gut homeostasis but also complement-related pathways such as C3 expression (Lian et al., 2016), systemic inflammatory tone (Yusufu et al., 2021), and brain Trp levels, which in turn affect emotional and behavioral regulation (Jenkins et al., 2016). The MGBA is thus not a vague metaphor but a biochemical network in which Trp metabolites are major messengers.

### 11.2 AHR is a context-dependent signal integrator in glia

Aryl hydrocarbon receptor was initially identified as a dioxin-binding factor, but it is now recognized as a conserved transcription factor with broad roles in aging, immunity, and CNS disease (Lee et al., 2017; Shinde and McGaha, 2018). It is expressed in neurons, microglia, and astrocytes (Rothhammer et al., 2016; Szychowski et al., 2016). This distribution makes AHR particularly relevant to AD, where glial interactions are central and systemic immune–metabolic signals increasingly intrude into brain biology.

A key insight from the literature is that AHR signaling is not uniformly anti-inflammatory. Its effect depends on ligand source, cell type, and inflammatory context. Astrocytic AHR generally restrains CNS inflammation: AHR-deficient astrocytes produce higher levels of pro-inflammatory mediators such as CCL2 and IL-6 and promote immune cell recruitment (Rothhammer et al., 2016). In neurons, AHR signaling influences ROS production, caspase-3 activation, and LDH release, indicating a role in stress responses and survival pathways (Szychowski et al., 2016).

Microglial AHR is more paradoxical. In the presence of exogenous or microbiota-derived AHR ligands, AHR can attenuate LPS-induced inflammatory outputs by reducing binding to the iNOS and TNF-α promoters and by inhibiting NF-κB-related signaling (Lee et al., 2015; Rothhammer et al., 2016). However, in the absence of adequate ligands, inflammatory stimuli may activate AHR through alternative routes, and AHR signaling can support pro-inflammatory microglial responses and neurotoxicity (Lee et al., 2015). This ligand-dependent inversion illustrates why the therapeutic implications of AHR signaling remain uncertain, and why caution is warranted before assigning this axis a decisive upstream role.

### 11.3 Trp metabolites as regulators of microglia–astrocyte communication

Emerging evidence suggests that Trp metabolites do not simply modulate glial cells independently; they influence their communication. Microbiota-derived metabolites such as IPA and I3A can activate microglial programs that subsequently signal to astrocytes through AHR-dependent pathways (Mu et al., 2018). Dietary Trp has been reported to induce microglial production of TGF-α and VEGF-B, which then modulate astrocytic transcriptional programs through AHR-mediated signaling (Otxoa-de-Amezaga et al., 2019). *Lactobacillus* and related bacterial taxa generate AHR-active metabolites that help maintain immune homeostasis (Ghiboub et al., 2020). *Limosilactobacillus reuteri*-derived indole metabolites can cross the BBB and interact with CNS AHR (Mu et al., 2018). These findings raise the possibility—though on the basis of indirect and largely non-AD evidence, and therefore remaining conjectural—that the quality and quantity of circulating Trp metabolites may influence whether microglia–astrocyte crosstalk remains regulatory or shifts toward inflammatory escalation.

**FIGURE 6:**
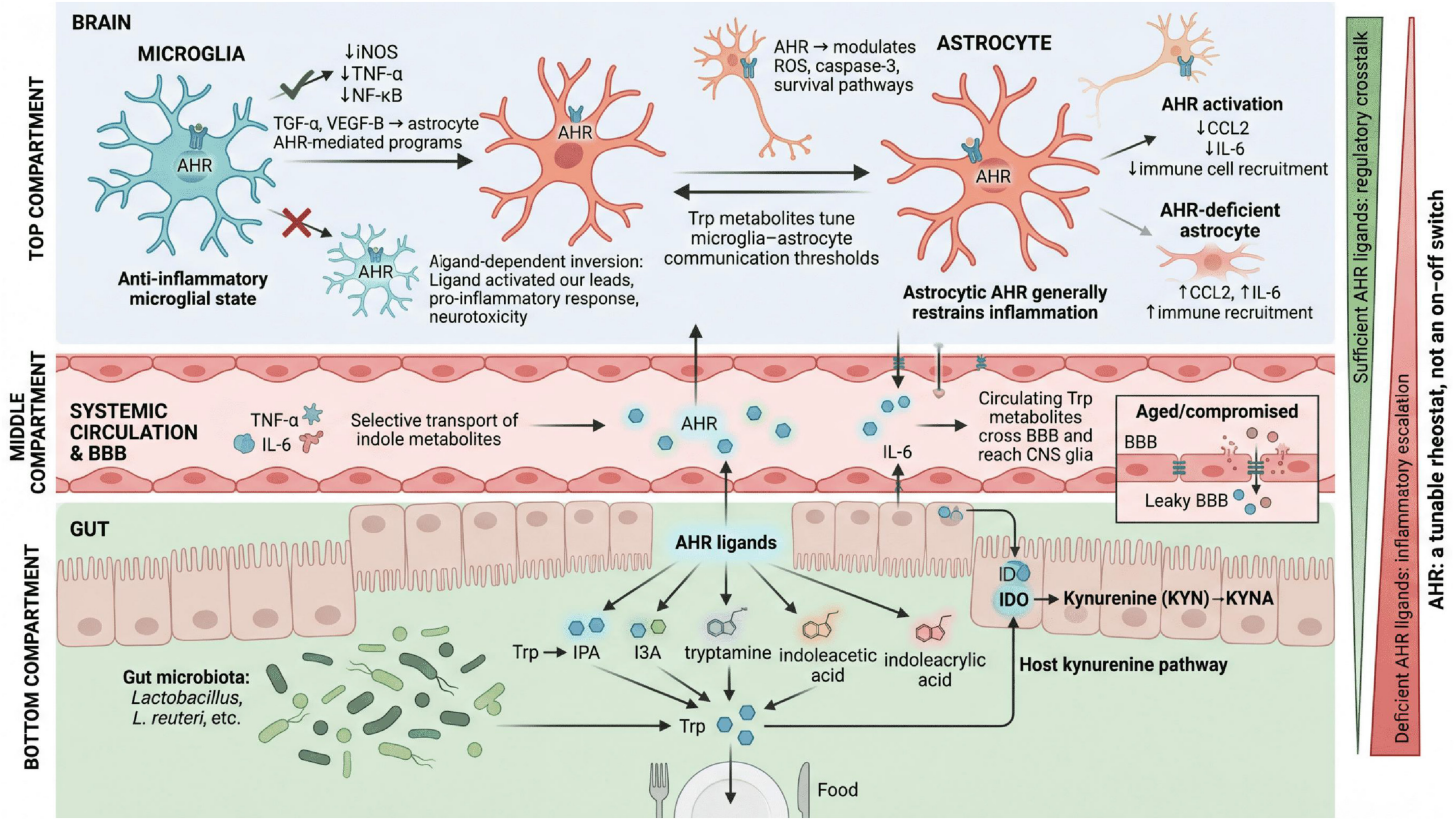
The tryptophan–microbiota– aryl hydrocarbon receptor (AHR) axis connects peripheral metabolism with central glial communication. (Bottom) Gut microbiota (*Lactobacillus*, *Limosilactobacillus reuteri*) convert dietary tryptophan into AHR ligands (IPA, I3A, tryptamine, indoleacetic/indoleacrylic acid); the host kynurenine pathway generates kynurenine (KYN) and kynurenic acid (KYNA) via indoleamine 2,3-dioxygenase (IDO). (Middle) Circulating tryptophan (Trp) metabolites cross the blood–brain barrier (BBB) (compromised with aging). (Top) In microglia, AHR with adequate ligands suppresses inducible nitric oxide synthase (iNOS)/tumor necrosis factor alpha (TNF-α)/nuclear factor kappa-light-chain-enhancer of activated B cells (NF-κB) and induces transforming growth factor alpha (TGF-α)/vascular endothelial growth factor B (VEGF-B) signaling to astrocytes; without ligands, AHR supports pro-inflammatory responses (ligand-dependent inversion). Astrocytic AHR restrains inflammation (↓CCL2, ↓IL-6); AHR deficiency promotes immune recruitment. Neuronal AHR modulates ROS and caspase-3. Right gradient bar: AHR functions as a tunable rheostat, not an on–off switch.

Tryptophan deficiency and dysregulated indoleamine 2,3-dioxygenase (IDO) activity further support this model. Trp-deficient diets induce gut dysbiosis and systemic inflammation (Yusufu et al., 2021), while Trp deficiency or IDO elevation activates NF-κB, oxidative stress, and pro-inflammatory responses (Robinson et al., 2006; Jiang et al., 2015). In a CNS already sensitized by aging, these changes may alter the ligand landscape available to glial AHR and thereby reprogram glial communication thresholds (Figure 6). We note, however, that most of this mechanistic chain—microbial ligand production, BBB transit, and cell-type-specific AHR signaling in glia—has been reconstructed from separate experimental systems rather than demonstrated as an integrated causal pathway in human AD, where direct evidence remains limited and heterogeneous.

### 11.4 Conflict point: AHR activation is unlikely to be universally therapeutic

Because AHR can suppress inflammatory programs, it is tempting to present AHR agonism as a straightforward therapeutic strategy. That conclusion is premature. The microglial literature clearly indicates that AHR signaling is conditional, especially with respect to ligand availability and inflammatory context (Lee et al., 2015). A ligand that produces protective astrocytic responses may not have identical effects in microglia or neurons. Conversely, insufficient ligand availability may convert AHR from a restraint mechanism into part of a pathological response. In practical terms, this means that “AHR activation” is not a single intervention. Its outcome will depend on which ligand is used, where it acts, at what disease stage, and in what broader metabolic environment.

This may partly explain why manipulating systemic inflammation or gut microbiota without considering CNS cell-state specificity often yields inconsistent results. The Trp–AHR axis should therefore be viewed as a tunable rheostat of glial crosstalk, not as a simple on–off anti-inflammatory switch.

## 12 A new synthesis: AD as a staged collapse of glial communication

**FIGURE 7:**
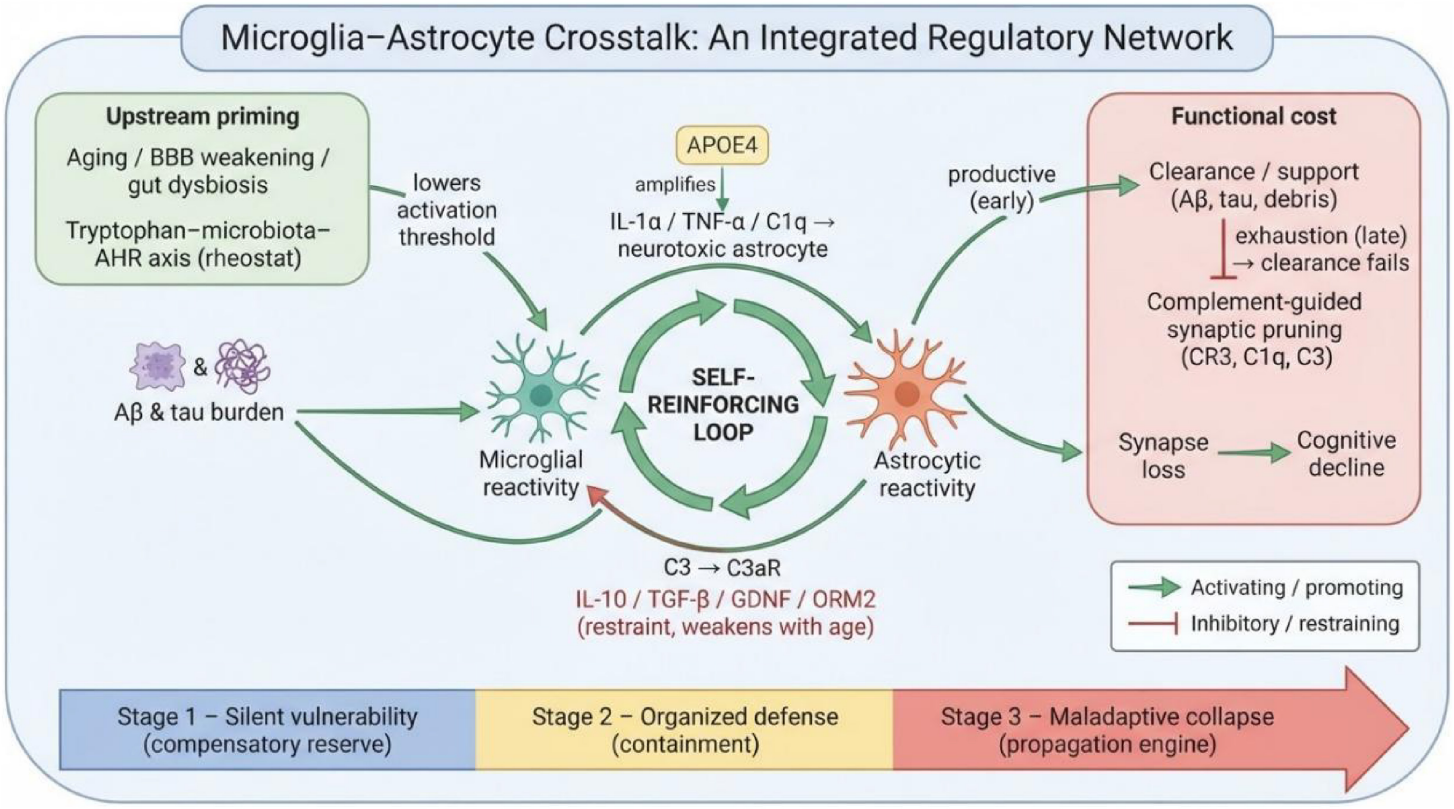
An integrated signed-network view of microglia–astrocyte crosstalk across the three disease stages. The mechanisms discussed throughout this review are consolidated into a single regulatory network, with nodes linked by signed interactions (green arrows, activating; red blunt-headed arrows, inhibitory). Aging, blood–brain barrier (BBB) weakening, gut dysbiosis, and the tryptophan–microbiota– aryl hydrocarbon receptor (AHR) axis lower the threshold for network activation, while rising amyloid-beta (Aβ) and tau burden drive both microglial and astrocytic reactivity. These engage in a bidirectional, self-reinforcing loop [microglialinterleukin-1 alpha (IL-1α)/tumor necrosis factor alpha (TNF-α)/complement component 1q (C1q) → neurotoxic astrocytes; astrocytic C3 → microglial complement C3a receptor (C3aR)], normally opposed by restraint signals [interleukin-10 (IL-10), transforming growth factor beta (TGF-β), GDNF, ORM2] that weaken with age and are further amplified by apolipoprotein E4 (APOE4). Downstream, glial reactivity supports clearance early but fails with sustained burden, while excessive complement-guided pruning (CR3, C1q, C3) drives the synapse loss that best correlates with cognitive decline. The bottom band maps these states onto the three stages: silent vulnerability, organized defense, and maladaptive collapse. The schematic is a qualitative integration of predominantly preclinical evidence, not a quantitative or validated model.

The evidence reviewed above supports a coherent model of AD in which a staged remodeling of microglia–astrocyte communication acts as an important amplifier of disease progression, operating in concert with—rather than instead of—the accumulation of toxic proteins, neuronal dysfunction, and vascular compromise (Figure 7).

In the preclinical stage, aging, vascular dysfunction, Trp-metabolic imbalance, and subtle gut microbial shifts alter the basal operating state of glia (Liddelow et al., 2017; Olah et al., 2018; Pan et al., 2025). Microglia become more easily primed; astrocytes lose some homeostatic buffering capacity; complement tone rises; lysosomal and metabolic reserves begin to erode. This stage may be clinically silent but biologically decisive because it sets the threshold for later pathology.

In the compensatory stage, Aβ deposition and early tau abnormalities trigger active glial cooperation. Microglia converge on plaques, astrocytes reorganize around lesions, complement is used to triage synapses and debris, and multiple crosstalk pathways attempt to limit spread (Wyss-Coray et al., 2003; Liu et al., 2017; Montoliu-Gaya et al., 2017). Trp-derived AHR ligands may help keep this state within adaptive bounds, especially in astrocytes and ligand-sufficient microglia (Rothhammer et al., 2016; Mu et al., 2018). During this phase, pathology is present but tissue organization still retains a degree of functional intelligence. Throughout these stages, anti-amyloid interventions that lower plaque burden may act primarily on the initiating input, whereas network hyperexcitability and vascular compromise continue to shape the tissue environment; this parallelism may help explain why amyloid removal slows, but does not arrest, decline in symptomatic patients (Sims et al., 2023; van Dyck et al., 2023).

In the maladaptive stage, chronic proteotoxic stress, persistent inflammatory exposure, impaired lysosomal degradation, complement overdrive, lipid disequilibrium, and loss of regulatory feedback transform compensatory crosstalk into self-amplifying loops (Wolfe et al., 2013; Xiao et al., 2014; Lee et al., 2022; Yang et al., 2024). Microglia remain reactive but become less discriminating and less degradatively competent. Astrocytes remain engaged but provide less support, release more complement and chemokines, and lose spatial and metabolic restraint. EV-mediated propagation broadens the pathological field (Goetzl et al., 2016; Drago et al., 2017). In this model, Aβ and tau no longer need to be the sole direct drivers of damage; the glial network itself may act as a propagation engine. We stress that this

“propagation engine” formulation is a conceptual proposal, not an empirically established property of the human disease; it has not been directly demonstrated longitudinally in humans.

This staged model suggests that the real point of no return in AD may be the moment when glial communication ceases to be corrective and becomes autonomously pathological. By the time that threshold is crossed, simply lowering Aβ burden may no longer restore functional tissue organization. This may be one reason why anti-Aβ therapies are most effective, if at all, early in the disease.

### 12.1 Operationalizing the framework: measurable variables, biomarker proxies, and falsifiable predictions

A legitimate concern is that terms such as “maladaptive loop” and “communication collapse” risk being descriptive rather than measurable. Building on the operational definitions introduced above, we specify here the variables, biomarker proxies, and directional predictions through which the framework can be tested and, in principle, refuted.

Measurable variables: The compensatory-to-maladaptive transition should be quantifiable along three axes. First, ratio-based molecular readouts: the tissue balance between microglial homeostatic and disease-associated programs (e.g., P2RY12/TMEM119 relative to APOE/TREM2 transcripts), and the astrocytic C3-to-clearance ratio (C3/GLT-1). Second, spatial-organizational metrics: plaque-coverage index, microglia–astrocyte nearest-neighbor distance within peri-plaque nets, and the degree of spatial co-organization of protective glial communities, all of which are now extractable from spatial transcriptomic and imaging datasets. Third, functional decoupling metrics: the relationship between a reactivity index and a clearance index, where a widening gap (rising reactivity, falling clearance) operationalizes “collapse.”

Fluid and imaging biomarker proxies in living patients: These tissue-level variables have accessible clinical surrogates. Astrocyte reactivity is indexed by plasma and CSF GFAP (Ferrari-Souza et al., 2022); microglial activation by sTREM2, YKL-40, and TSPO-PET; complement tone by CSF C1q, C3, and C4; and AHR-ligand sufficiency by CSF/plasma indole and kynurenine metabolite levels. Critically, we propose that trajectories and ratios, rather than single static values, index communication state: a declining sTREM2/GFAP ratio, or a rising GFAP against a plateauing sTREM2, would mark the transition from a microglia-supported compensatory state to an astrocyte-reactivity-predominant maladaptive state.

Falsifiable predictions: The framework generates the following testable predictions, several of which are already partly addressable with existing cohorts: (1) In longitudinal biomarker cohorts, a declining sTREM2/GFAP ratio (or GFAP rising while sTREM2 plateaus/declines) will predict accelerated cognitive and tau-PET progression independently of amyloid burden. If reactivity markers instead rose in fixed proportion to one another and to amyloid across all stages, with no decoupling, the staged-collapse model would be falsified (Pascoal et al., 2021; Bellaver et al., 2023). (2) Single-node interruption (e.g., C3aR antagonism, C1q blockade) will reduce synapse loss and cognitive decline only when applied before the loop becomes self-sustaining; efficacy should diminish sharply once biomarker evidence of decoupling is present. Failure of stage-dependence would weaken the “loop” construct. (3) Restoring astrocytic GLT-1/EAAT2 will produce a quantifiable reduction in synaptic C1q colocalization and microglial synaptic engulfment—a specific, directional, and refutable prediction. (4) AHR-directed intervention will shift glial state toward the regulatory pole only in individuals with measurable ligand deficiency (low circulating indole metabolites); it should be neutral or harmful in ligand-sufficient individuals. Uniform effects regardless of baseline ligand status would falsify the “rheostat” model.

Framing the framework in these terms converts it from a narrative into a set of measurable variables and directional hypotheses that can be corroborated or refuted with spatial-omics, longitudinal fluid biomarkers, and stage-stratified interventional studies.

### 12.2 Limitations of the framework and open questions

While we have argued that microglia–astrocyte crosstalk functions as an organizing principle of AD, intellectual honesty requires acknowledging that this framework remains a hypothesis supported largely by associative and preclinical evidence rather than by direct causal demonstration in humans.

First, much of the mechanistic data reviewed here derives from rodent models—including APP/PS1, P301S, and related transgenic lines—that recapitulate individual features of AD but do not reproduce the full spatiotemporal complexity of the sporadic human disease (Drummond and Wisniewski, 2017; Sasaguri et al., 2017). Species differences in microglial and astrocytic transcriptional programs, complement biology, and AHR ligand handling mean that findings such as C3–C3aR-driven synapse loss or Plexin-B1-dependent glial positioning cannot be assumed to translate directly to human pathophysiology.

Second, the human evidence supporting a communication-centered model is predominantly correlative. Proteomic co-expression networks, spatial transcriptomics, and single-nucleus RNA sequencing reveal that reactive microglia, astrocytes, and vulnerable neurons occupy structured microenvironments and that glial interaction signatures change with disease stage. However, these are cross-sectional, postmortem snapshots. They establish association, not causation, and they cannot resolve whether distorted glial communication drives neurodegeneration, arises as a consequence of it, or reflects a shared upstream process.

Third, the “staged collapse” model implies a temporal sequence—silent vulnerability, compensation, and maladaptive collapse—that has not been directly demonstrated within individual patients over time. The boundaries between these stages are conceptual, and it remains unclear whether the transition to a self-sustaining pathological state represents a genuine biological threshold or a gradual continuum.

Fourth, technical limitations constrain interpretation. Tissue dissociation and nuclear isolation can introduce artifacts that distort glial state assignments (Marsh et al., 2022); ligand–receptor inference from transcriptomic data does not confirm functional signaling; and the causal role of the Trp–microbiota–AHR axis in human AD currently rests on a small and heterogeneous body of clinical evidence.

Finally, the framework’s central claim—that AD is “organized” at the level of communication rather than isolated molecular nodes—is difficult to test with current tools. Definitive support would require longitudinal human biomarkers of glial communication states, cell-type-specific and stage-specific causal perturbation in models that better approximate human biology, and demonstration that restoring communication homeostasis alters clinical trajectory. Until such evidence exists, microglia–astrocyte crosstalk is best regarded as a compelling integrative hypothesis that reframes existing observations, rather than an established causal principle. We present it in that spirit: as a framework intended to generate testable predictions and guide the next generation of mechanistic and therapeutic studies.

### 12.3 Human relevance and translational anchoring of the framework

Although much of the mechanistic detail reviewed above derives from experimental models, several independent lines of human evidence anchor the communication-centered framework and indicate how it may translate to clinical disease.

At the genetic level, human association studies have repeatedly converged on glial and innate-immune pathways rather than neuron-autonomous ones. Common and rare variants in APOE, TREM2, CR1, CLU, MS4A, and complement-related loci are among the strongest genetic determinants of AD risk, and the largest genome-wide analyses to date implicate microglial and complement biology as core disease pathways (Bellenguez et al., 2022). Because several of these molecules—APOE, TREM2, and complement components—are precisely the nodes we identify as mediators of microglia–astrocyte crosstalk, human genetics provides causal, disease-relevant support that the glial communication network is not merely a bystander.

At the tissue level, human postmortem single-nucleus and spatial transcriptomic studies demonstrate that reactive microglia, disease-associated astrocytes, and vulnerable neurons form structured multicellular communities whose organization tracks with disease stage and cognitive status (Cain et al., 2023; Gabitto et al., 2024; Green et al., 2024). Proteomic co-expression analyses further show that astrocyte- and microglia-specific modules are abnormally coupled early in the human disease continuum (Liu et al., 2024), and human neuropathological work indicates that glial phenotypic changes can precede overt tangle formation and predict cognitive status at intermediate Braak stages (Taddei et al., 2022, 2023). These observations, while cross-sectional, indicate that the spatial and relational logic central to our model is a genuine feature of human AD tissue.

Most importantly for translation, glial communication states are increasingly measurable in living patients. Fluid biomarkers of astrocyte reactivity (plasma and CSF GFAP) and of microglial activation (soluble TREM2, YKL-40) change dynamically across the AD continuum and correlate with amyloid, tau, and cognitive trajectories (Suárez-Calvet et al., 2016; Benedet et al., 2021). *In vivo* TSPO positron emission tomography allows regional neuroinflammation to be imaged longitudinally in patients (Hamelin et al., 2016). Together, these tools raise the realistic prospect of stratifying patients according to the dominant glial communication state—for example, astrocyte-reactivity-predominant versus microglial-activation-predominant profiles—and of monitoring whether an intervention shifts that state.

We therefore propose that the practical value of this framework lies in its testability in humans: existing and emerging biomarkers (plasma GFAP, sTREM2, complement-related signatures, and neuroinflammation imaging), combined with genetic stratification, could be used to determine whether individual patients occupy a compensatory or a maladaptive communication state, and to match protein-directed and communication-directed therapies accordingly. Framed this way, microglia–astrocyte crosstalk is not only a conceptual synthesis but a source of concrete, clinically measurable hypotheses.

## 13 Therapeutic implications: from suppressing inflammation to reprogramming communication

If AD is in large part a disease of maladaptive microglia–astrocyte crosstalk, then therapeutic logic must shift accordingly. Several principles follow.

First, blanket suppression of glial activation is unlikely to succeed. Microglia and astrocytes perform essential protective functions in early disease, including plaque compaction, debris clearance, barrier formation, and trophic suppor t (Gomez-Arboledas et al., 2018; Sanchez-Mico et al., 2021). Eliminating these responses indiscriminately may worsen tissue vulnerability even if some inflammatory markers decline.

Second, therapies should be cell type-specific and disease stage-specific. TREM2 agonism may improve Aβ-related microglial functions (Wang et al., 2015; Parhizkar et al., 2019; Schlepckow et al., 2020; Zhao et al., 2022), but its consequences in tauopathy appear more complex (Leyns et al., 2017; Sayed et al., 2018). Complement inhibition may protect synapses, yet excessive blockade could interfere with beneficial clearance and remodeling (Dejanovic et al., 2018; Wu et al., 2019). Astrocyte-targeted strategies that enhance lysosomal biogenesis or restore GLT-1 expression may reduce Aβ burden and synaptic dysfunction without abolishing astrocytic support functions (Wu et al., 2020). Similarly, modulating IL-3/IL-3R, ORM2, PAI-1, or other crosstalk mediators may prove more precise than broad anti-inflammatory suppression (Liu R.-M. et al., 2011; Akhter et al., 2018).

Third, the Trp–microbiota–AHR axis should be explored as an upstream intervention platform. This could include dietary strategies, microbiota reshaping, selective augmentation of beneficial indole-producing taxa, or ligand-biased AHR modulation (Jin et al., 2014; Ma et al., 2020). However, given the context-dependent effects of AHR, such approaches will require careful attention to ligand identity, pharmacokinetics, BBB penetration, and cell type specificity (Rothhammer et al., 2016; Szychowski et al., 2016). A promising avenue may lie in restoring a physiological ligand environment rather than applying strong synthetic activation indiscriminately.

Fourth, future biomarker efforts should move beyond static protein burden and toward signatures of glial communication states. Spatial transcriptomics, snRNA-seq, EV profiling, complement-related signatures, ligand–receptor interaction maps, and metabolic readouts of Trp/AHR signaling could together define a more informative disease atlas (Smith et al., 2022; Liu et al., 2024). Such biomarkers may reveal whether a patient’s pathology is dominated by failed clearance, complement-mediated synapse loss, astrocytic inflammatory conversion, ligand-deficient AHR signaling, or glial metabolic exhaustion. Precision therapy in AD will likely require that level of granularity.

It is important to distinguish which of these strategies have advanced to human testing from those that remain preclinical, as the two carry very different levels of evidentiary support. Among communication-relevant approaches, the most clinically mature are those acting on the microglial receptor TREM2 and on systemic metabolic-inflammatory signaling, and both have now yielded definitive—largely negative—Phase 2/3 readouts that sharpen rather than resolve the questions this review raises.

TREM2 agonistic antibodies designed to enhance protective microglial functions have progressed to clinical evaluation. A phase 2, randomized, double-blind, placebo-controlled trial of the humanized TREM2 agonistic monoclonal antibody AL002 was conducted in 381 participants with early AD, randomized to receive AL002 or placebo intravenously every 4 weeks for 48–96 weeks. The results showed that AL002 demonstrated sustained target engagement and pharmacodynamic responses in the central nervous system, as demonstrated by reductions in soluble TREM2 and increases in osteopontin in cerebrospinal fluid, respectively, but the study did not meet the primary endpoint of change from baseline in the Clinical Dementia Rating-Sum of Boxes score versus placebo, with the most frequent treatment-emergent adverse events being magnetic resonance imaging changes resembling amyloid-related imaging abnormalities (ARIA). The investigators concluded that this first trial of a TREM2 agonistic antibody in early AD was negative but provides findings relevant to the study of TREM2 therapeutics and ARIA (Mummery et al., 2026). This outcome directly underscores the stage- and context-dependence emphasized throughout this review: robust target engagement in the CNS was not sufficient to translate into clinical or biomarker benefit, suggesting that agonizing TREM2 in established, symptomatic disease may be too late a stage for this particular node of microglial communication to be therapeutically decisive.

Glucagon-like peptide-1 receptor agonists, which attenuate the microglia-to-astrocyte neurotoxic conversion axis in preclinical models, were first shown to be neuroprotective through agonism of microglial glucagon-like peptide-1 receptor (GLP1R) using a brain-penetrant peptide, which prevents the generation of neurotoxic astrocytes and ameliorates disease progression in two rodent models of Parkinson’s disease (Yun et al., 2018)—the mechanism underlying NLY01. This mechanistic rationale motivated large-scale human testing: the evoke and evoke+ trials were multicentre, randomized, double-blind, placebo-controlled phase 3 trials conducted across 566 sites in 40 countries, assessing the efficacy and safety of oral semaglutide up to 14 mg once daily in participants with amyloid-confirmed AD, aged 55–85 years, with mild cognitive impairment or mild dementia, randomly assigned to once-daily semaglutide or placebo for up to 156 weeks. The definitive results showed that mean changes in CDR-SB score from baseline to week 104 were similar between semaglutide and placebo groups in both trials, with no significant estimated treatment difference, leading the authors to conclude that oral semaglutide was not efficacious in slowing clinical progression in participants with early AD (Cummings et al., 2025, 2026). This dissociation between biomarker modulation and clinical benefit is directly relevant to the communication-based framework proposed here: it suggests that attenuating peripheral or central inflammatory signaling alone, without concurrently engaging the more proximal astrocytic and complement-mediated effector mechanisms discussed above, may be insufficient to alter clinical trajectory once neurodegeneration is established.

Approaches targeting the microbiota–gut–brain axis have also reached the clinic: GV-971, a sodium oligomannate that has demonstrated solid and consistent cognition improvement in a phase 3 clinical trial in China, suppresses gut dysbiosis and the associated phenylalanine/isoleucine accumulation, harnesses neuroinflammation and reverses the cognition impairment (Wang et al., 2019), offering a partial clinical precedent for the Trp–microbiota–AHR framework advanced here.

By contrast, most of the crosstalk-specific interventions we highlight remain preclinical. Complement-directed strategies (C1q or C3/C3aR blockade) have shown protection in mouse models but have not yet been validated in AD patients, and their clinical development is largely confined to other complement-driven CNS and retinal diseases. Astrocyte-directed strategies—restoration of GLT-1/EAAT2, enhancement of astrocytic lysosomal biogenesis, and modulation of IL-3/IL-3R, ORM2, or PAI-1—are supported only by experimental data. Likewise, direct pharmacological targeting of the AHR with defined ligands, and selective augmentation of indole-producing bacterial taxa, remain at the preclinical stage and face the ligand-, cell- type-, and stage-specific complexities discussed above.

Recognizing this translational gap is essential. The recent negative Phase 2/3 outcomes for both AL002 and semaglutide—despite confirmed target engagement and favorable biomarker shifts, respectively—reinforce rather than undermine the central argument of this review: single-node interventions directed at one arm of glial communication, tested largely in symptomatic patients, may arrive too late or address too narrow a mechanism to alter clinical course. The framework we propose is intended not to overstate therapeutic readiness, but to prioritize which communication nodes most urgently warrant translation—potentially earlier in the disease continuum and in combination—and to argue that these should be tested in combination with, rather than instead of, established protein-directed therapies.

Finally, anti-Aβ or anti-tau therapies should not be abandoned but repositioned within a broader strategy. The demonstrated, biomarker-confirmed clinical efficacy of lecanemab and donanemab establishes protein removal as a validated therapeutic pillar and an essential backbone for combination approaches (Sims et al., 2023; van Dyck et al., 2023), not a strategy to be replaced. Protein-directed interventions may reduce the initiating burden, but they are unlikely to normalize brain function unless combined with measures that restore the logic of glial communication. In this view, the future of AD therapy is not “protein versus inflammation.” It is protein control plus communication repair.

## 14 From framework to actionable strategy: stage-specific targets, biomarkers, and interventions

A conceptual framework earns clinical relevance only if it specifies what to measure, what to target, and when to intervene. Here we translate the three-stage model of glial-communication failure (section “12 A new synthesis: AD as a staged collapse of glial communication”) into concrete, testable proposals. We emphasize that these are hypotheses intended to structure clinical investigation, and several rest on mechanistic inference from model systems rather than on human data; none should be interpreted as clinical guidance.

### 14.1 Candidate therapeutic targets

The framework nominates the molecular nodes of microglia–astrocyte crosstalk as tractable targets. These include: (i) the microglial TREM2–APOE signaling axis, where TREM2 agonism aims to sustain the protective, plaque-compacting microglial state (Yuan et al., 2016); (ii) complement-mediated synapse elimination, targetable via C1q or C3 blockade to limit maladaptive pruning (Hong et al., 2016); (iii) reactive-astrocyte conversion, where suppression of the neurotoxic (C3^+^/A1-like) phenotype—e.g., by interrupting IL-1α/TNF/C1q induction—may preserve homeostatic support (Liddelow et al., 2017); (iv) astrocytic glutamate handling (EAAT2/GLT-1 restoration) to contain excitotoxicity and network hyperexcitability (Lin et al., 2012); and (v) NLRP3 inflammasome and downstream IL-1β signaling as an amplifier common to both cell types (Heneka et al., 2015). Critically, the framework predicts that the same target may be beneficial or harmful depending on disease stage—for example, microglial activation is protective early but detrimental once communication has decompensated—which reframes prior contradictory trial results (section “13 Therapeutic implications: from suppressing inflammation to reprogramming communication”).

### 14.2 Stage-specific intervention logic

The central actionable claim is that intervention timing must be matched to the state of the glial network rather than to symptom severity alone (Table 1). In the compensatory stage, when microglial and astrocytic responses remain coordinated, protein-directed therapy (anti-Aβ antibodies) combined with support of protective glial function is predicted to yield maximal benefit. In the transitional stage, marked by rising neuroinflammatory signaling and early network hyperexcitability, the priority shifts to restraining maladaptive inflammation (e.g., NLRP3/IL-1β inhibition, complement modulation) while function is still recoverable. In the decompensated stage, broad immunosuppression is predicted to be ineffective or harmful, and realistic goals shift toward network stabilization and symptomatic support. This logic directly explains why glia-directed agents such as AL002 (Mummery et al., 2026) and repurposed anti-inflammatory drugs may fail when administered without stage stratification.

**TABLE 1:** Stage-specific mapping of the glial-communication framework to candidate biomarkers, therapeutic priorities, and predicted therapeutic response.

Stage	Dominant glial state	Candidate biomarkers (fluid/imaging)	Proposed therapeutic priority	Predicted response to protein-directed therapy
Compensatory	Coordinated protective microglia + homeostatic astrocytes	↑ sTREM2 with low NfL; low GFAP/NfL ratio; preserved fMRI connectivity; focal TSPO-PET	Anti-Aβ therapy + preserve protective glial function (TREM2 agonism)	Maximal benefit
Transitional	Rising neuroinflammation; emerging astrocyte reactivity; network hyperexcitability	Rising GFAP, YKL-40, IL-1β; increasing GFAP/NfL and falling sTREM2/NfL; EEG/fMRI hyperexcitability	Restrain maladaptive inflammation (NLRP3/IL-1β, complement C1q/C3 modulation); protect synapses	Partial; requires combination
Decompensated	Loss of coordination; neurotoxic reactive astrocytes; net synapse loss	High NfL; high GFAP with declining sTREM2; widespread TSPO/astrocyte-PET; disrupted connectivity	Network stabilization and symptomatic support; broad immunosuppression predicted ineffective/harmful	Minimal

### 14.3 Clinically applicable biomarkers

The framework is falsifiable because it maps onto measurable readouts already entering clinical use. Fluid markers of glial state—CSF and plasma sTREM2, GFAP, YKL-40 (CHI3L1), and complement components—together with the established markers of proteinopathy (plasma p-tau217, Aβ42/40) and neurodegeneration (NfL), can be combined to stage the glial network rather than the protein burden alone. We propose that the ratio and trajectory of glial-activation to neurodegeneration markers (e.g., GFAP or sTREM2 relative to NfL) may index the transition between compensatory and decompensated states more sensitively than any single analyte (Valletta et al., 2025). Imaging correlates include TSPO-PET (microglial activation), reactive-astrocyte PET tracers (e.g., MAO-B/deprenyl-based), and MRI/fMRI measures of network hyperexcitability and connectivity. A concrete, testable prediction is that individuals with high glial-activation markers but preserved connectivity occupy the therapeutic window in which glia-directed intervention will be most effective.

## 15 Conclusion

Alzheimer’s disease should no longer be understood solely as a disorder of Aβ deposition and tau aggregation. It is also, and importantly at the stage of clinical decline, a disease of failing intercellular coordination. Microglia and astrocytes normally operate as a homeostatic partnership that stabilizes synapses, supports neuronal metabolism, controls immune responses, and preserves tissue architecture. In aging and AD, that partnership is progressively rewired. Protective communication gives way to distorted signaling loops involving complement, chemokines, cytokines, trophic factors, EVs, lipid mediators, and metabolic stress pathways. Once these loops become self-sustaining, they no longer merely respond to pathology—they actively drive it.

Placing microglia–astrocyte crosstalk at the center of AD pathogenesis yields a more integrative explanation for the coexistence of plaque growth, tau spread, neuroinflammation, synapse loss, and circuit dysfunction. It also clarifies why therapeutic efforts aimed at only one lesion or one inflammatory mediator have frequently disappointed. Much of the disease’s tissue-level organization may be shaped at the level of communication networks, not solely by isolated molecular nodes.

Within this framework, the Trp–microbiota–AHR axis represents one plausible but still unproven upstream input that merits further investigation rather than an established regulator. Although direct evidence in human AD is currently limited and its effects are context-dependent and bidirectional, by linking diet, gut microbial metabolism, peripheral immune tone, and glial transcriptional programs it could, in principle, influence the threshold at which microglia–astrocyte communication shifts from adaptive to maladaptive. The major lesson here is not that AHR should simply be activated, but that glial signaling pathways must be interpreted through the lenses of ligand specificity, disease timing, and cellular context.

The next phase of AD research should therefore move from describing glial “activation” toward decoding glial communication states. Crucially, this shift is empirically tractable rather than merely conceptual: the framework generates falsifiable, clinically testable predictions: that glial-to-neurodegeneration biomarker ratios stage the disease more informatively than protein burden alone; that the efficacy of both protein- and glia-directed therapies is stage-dependent; and that a definable therapeutic window—high glial activation with preserved connectivity—identifies patients most likely to benefit from glia-directed intervention. These predictions can be tested in existing longitudinal and trial cohorts, providing a direct route from concept to clinical evaluation. The essential translational challenge is not to silence microglia or astrocytes, but to restore the conditions under which they can once again cooperate productively. If that shift in perspective is embraced, the field may finally move beyond the false choice between proteinopathy and inflammation and toward a more realistic biology of neurodegeneration.
